# Insights into Structure-Activity Relationships of 3-Arylhydrazonoindolin-2-One Derivatives for Their Multitarget Activity on β-Amyloid Aggregation and Neurotoxicity

**DOI:** 10.3390/molecules23071544

**Published:** 2018-06-26

**Authors:** Rosa Purgatorio, Modesto de Candia, Annalisa De Palma, Francesco De Santis, Leonardo Pisani, Francesco Campagna, Saverio Cellamare, Cosimo Damiano Altomare, Marco Catto

**Affiliations:** 1 Dipartimento di Farmacia-Scienze del Farmaco, Università degli Studi di Bari “Aldo Moro”, via E. Orabona 4, I-70125 Bari, Italy; rosa.purgatorio@uniba.it (R.P.); modesto.decandia@uniba.it (M.d.C.); leonardo.pisani@uniba.it (L.P.); francesco.campagna51@gmail.com (F.C.); saverio.cellamare@uniba.it (S.C.); cosimodamiano.altomare@uniba.it (C.D.A.); 2 Dipartimento di Bioscienze, Biotecnologie e Biofarmaceutica, Università degli Studi di Bari “Aldo Moro”, via E. Orabona 4, I-70125 Bari, Italy; annalisa.depalma@uniba.it (A.D.P.); desantis.nutrizione@gmail.com (F.D.S.)

**Keywords:** beta-amyloid aggregation inhibitors, indolin-2-ones, Alzheimer’s disease, quantitative structure-activity relationships, multitarget activity

## Abstract

Despite the controversial outcomes of clinical trials executed so far, the prevention of β-amyloid (Aβ) deposition and neurotoxicity by small molecule inhibitors of Aβ aggregation remains a target intensively pursued in the search of effective drugs for treating Alzheimer’s disease (AD) and related neurodegeneration syndromes. As a continuation of previous studies, a series of new 3-(2-arylhydrazono)indolin-2-one derivatives was synthesized and assayed, investigating the effects of substitutions on both the indole core and arylhydrazone moiety. Compared with the reference compound **1**, we disclosed equipotent derivatives bearing alkyl substituents at the indole nitrogen, and fairly tolerated bioisosteric replacements at the arylhydrazone moiety. For most of the investigated compounds, the inhibition of Aβ_40_ aggregation (expressed as pIC_50_) was found to be correlated with lipophilicity, as assessed by a reversed-phase HPLC method, through a bilinear relationship. The *N*^1^-cyclopropyl derivative **28** was tested in cell-based assays of Aβ_42_ oligomer toxicity and oxidative stress induced by hydrogen peroxide, showing significant cytoprotective effects. This study confirmed the versatility of isatin in preparing multitarget small molecules affecting different biochemical pathways involved in AD.

## 1. Introduction

Alzheimer’s disease (AD) is the most common cause of age-related neurodegenerative pathologies. AD represents a serious challenge for health systems, physicians and caregivers, because of its disabling course and the limited efficacy of pharmacological therapies [1]. The treatments approved for AD, namely the restoration of cholinergic transmission by means of acetylcholinesterase inhibitors [2], and the neuroprotection from glutamate excitotoxicity exerted by memantine [3], are only symptomatic and do not meet the clinical need for effective disease-modifying drugs.

A typical feature of AD consists in the deposition of extracellular β-amyloid (Aβ) peptide aggregates (amyloid plaques), starting from cholinergic neurons of hippocampus and then progressively extending to the whole brain cortex [4]. Aβ peptide derives from proteolysis of amyloid precursor protein (APP) catalyzed by β- and γ-secretases, and is formed as 40- (Aβ_40_) or 42-mer (Aβ_42_). Aβ monomers in AD brains aggregate to soluble oligomers, that in turn lead to intermediate protofibrils and finally to the deposition of amyloid plaques [5]. The formation of such oligomeric and prefibrillar species is correlated with the neurotoxicity in the AD brain, which represents a major causal factor of the cognitive impairment and the synaptic loss in AD patients [6,7,8].

**Figure 1:**
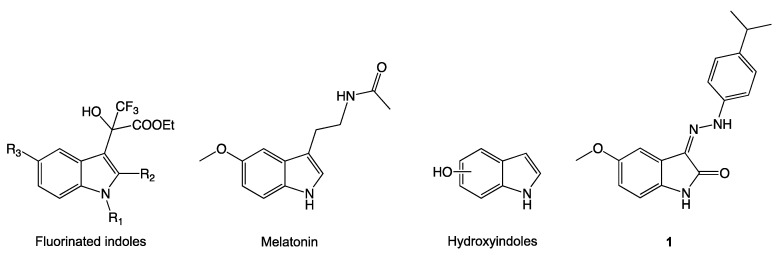
Structures of indole derivatives endowed with amyloid anti-aggregating properties.

The amyloidogenesis of Aβ occurs since the early stages of the disease insurgence, so that preventing the oligomerization and/or fibrillization process could represent a promising disease-modifying treatment for AD. Despite the number of research findings in this field, only a very small number of molecules have reached the preclinical stage, and no one entered therapy so far [9]. However, many small molecules acting as disruptors of protein-protein interactions have demonstrated potential in inhibiting Aβ aggregation [10]. Among them, indole derivatives such as melatonin [11], fluorinated indoles [12], hydroxyindoles [13] (Figure 1) displayed the structural features for an efficient antiaggregating activity. Particularly, they act as intercalators in hydrophobic interactions between Aβ side chains, including aromatic π-stacking interactions [14,15,16,17]. As recently shown by our structure-activity relationship (SAR) studies [16,18], these hydrophobic interactions taking place between Aβ and many classes of small molecules could be reinforced by polar interactions and/or hydrogen bond (HB) formation.

**Figure 2:**
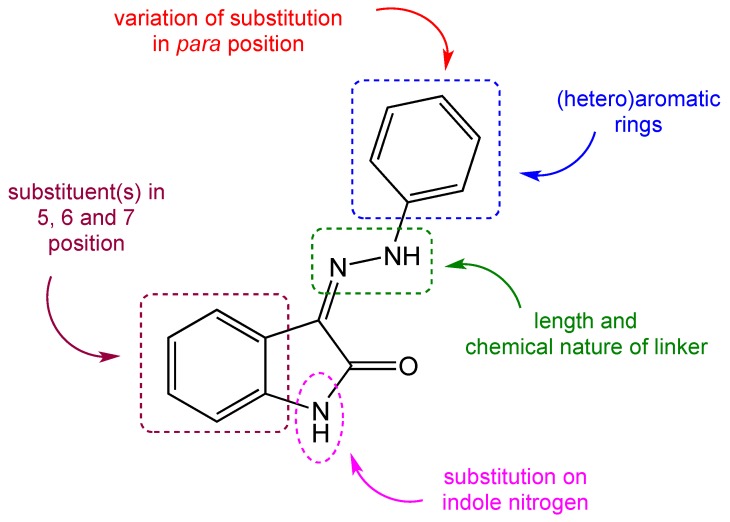
Summary of the investigated structural modifications of isatin-3-arylhydrazones.

**Scheme 1:**
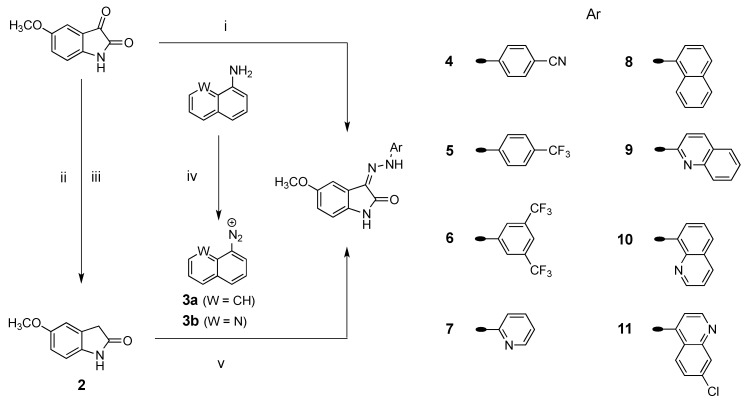
*Reagents and conditions*: (**i**) ArNHNH_2_, MeOH, room temperature; (**ii**) NH_2_NH_2_∙H_2_O, EtOH; (**iii**) NaOH, EtOH, H_2_O, reflux; (**iv**) NaNO_2_, HCl, 0 °C; (**v**) MeOH, CH_3_COONa, 0 °C.

**Scheme 2:**
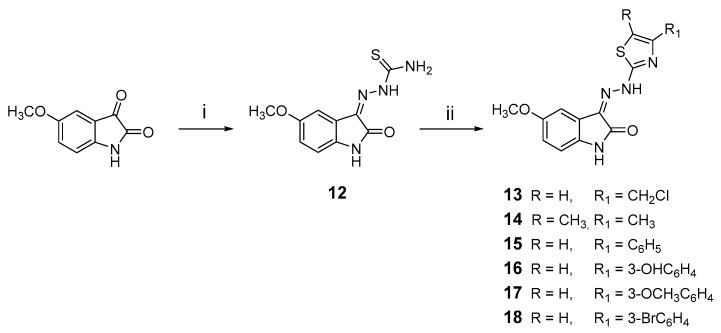
*Reagents and conditions*: (**i**) thiosemicarbazide, isopropanol, room temperature; (**ii**) (bromomethyl) alkyl or aryl ketone, EtOH, room temperature.

**Scheme 3:**
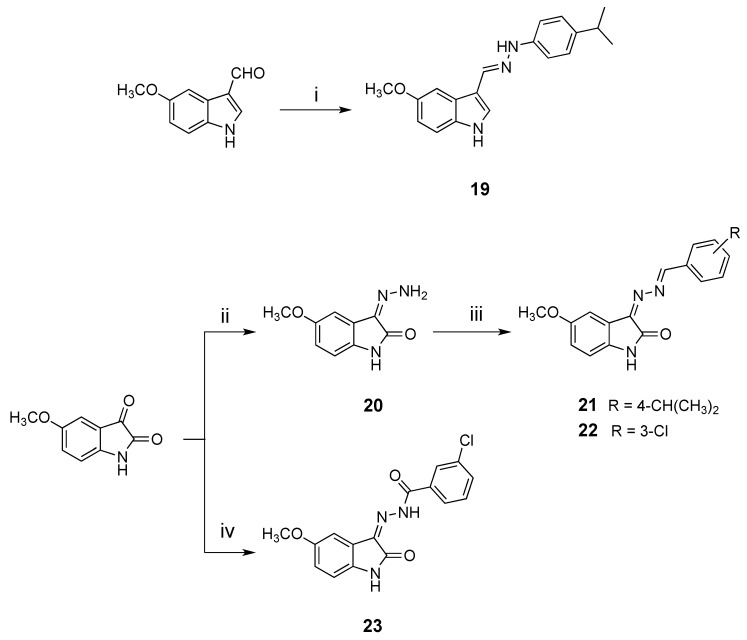
*Reagents and conditions*: (**i**) 4-isopropylphenylhydrazine hydrochloride, MeOH, room temperature; (**ii**) NH_2_NH_2_, AcOH, reflux; (**iii**) R-C_6_H_4_CHO, MeOH, reflux; (**iv**) 3-chlorobenzohydrazide, MeOH, room temperature.

**Scheme 4:**
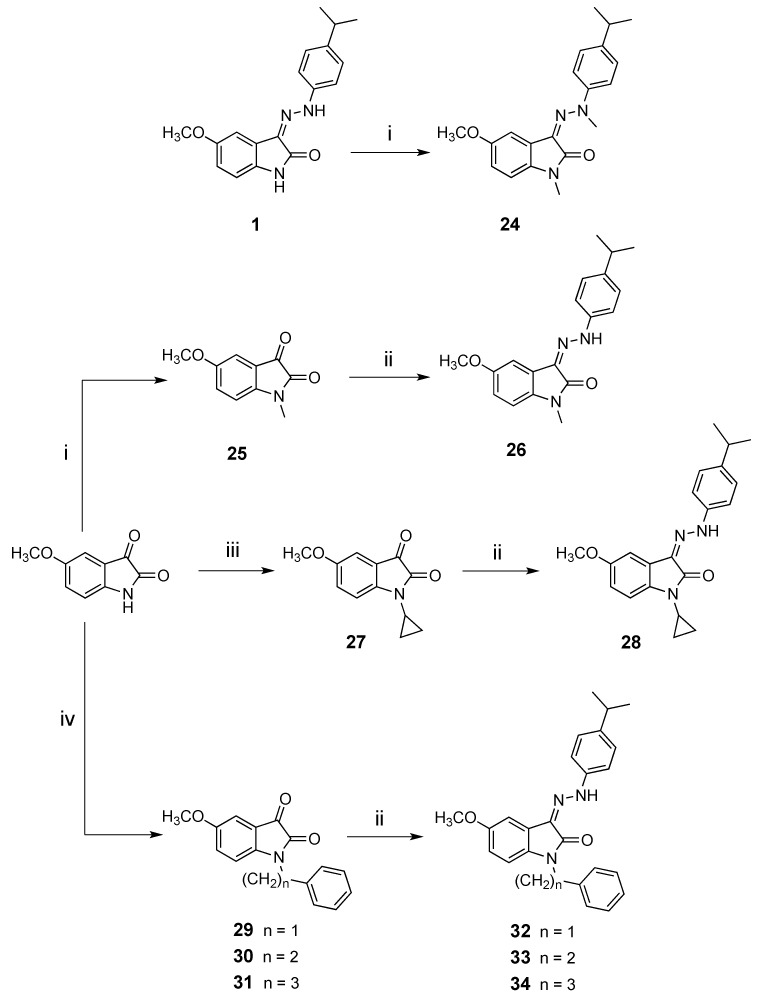
*Reagents and conditions*: (**i**) Methyl iodide, K_2_CO_3_, DMSO, room temperature; (**ii**) 4-isopropylphenylhydrazine hydrochloride, MeOH, room temperature; (**iii**) Cyclopropylboronic acid, Na_2_CO_3_, Cu(OAc)_2_, 1,2-dichloroethane, bipyridine, 50 °C; (**iv**) Br(CH_2_)_n_C_6_H_5_, K_2_CO_3_, DMSO, room temperature.

**Scheme 5:**
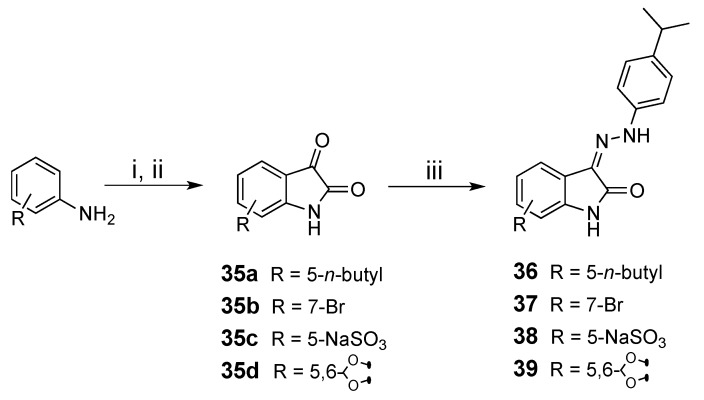
*Reagents and conditions*: (**i**) CCl_3_CH(OH)_2_, Na_2_SO_4_∙10H_2_O, H_2_O, HCl conc., NH_2_OH∙HCl, reflux; (**ii**) H_2_SO_4_ conc., 80 °C; (**iii**) 4-isopropylphenylhydrazine hydrochloride, MeOH, room temperature.

From previous studies [19,20,21,22], 5-methoxyisatin 3-(4-isopropylphenyl)hydrazone (**1**, Figure 1) was identified as a promising inhibitor of Aβ aggregation and acetylcholinesterase (AChE), with IC_50_s in the submicromolar and low μM range, respectively. Herein, we synthesized a number of congeners of compound **1**, and evaluated the in vitro activity as inhibitors of Aβ_40_ aggregation. With the aim of extending the SAR exploration, the new derivatives were designed in order to undertake a more systematic exploration (Figure 2) of: (i) the 3-arylhydrazone moiety, by introducing a number of diverse substituents and/or modifying the aryl substituent (compounds **4**–**11**, Scheme 1; compounds **13**–**18**, Scheme 2); (ii) the length and the chemical nature of the linker (compounds **19** and **21**–**23**, Scheme 3); (iii) the substitution either on the nitrogen (compounds **24**, **26**, **28**, **32**–**34**; Scheme 4) and the benzene moiety of the indole ring (compounds **36**–**39**, Scheme 5). SARs, including correlation of antiaggregating activity with lipophilicity, were investigated, and for one of the most potent Aβ_40_ aggregation inhibitors (compound **28**) cytoprotection from toxic Aβ_42_ oligomers in a cell-based assay was evaluated. Moreover, taking into account the role of oxidative stress in AD and other neurodegenerative diseases [23], the antioxidant property of **28** against hydrogen peroxide insult in SH-SY5Y cells was also tested.

## 2. Results and Discussion

### 2.1. Chemistry

New 5-methoxy-3-arylhydrazonoindolin-2-ones **4**–**11** were prepared by condensation of 5-methoxyisatin with the corresponding (hetero)arylhydrazines (compounds **4**–**7**, **9**, **11**) or alternatively via azo coupling of aryldiazonium salts **3a**,**b **with 5-methoxyindolin-2-one **2** [24] to yield the compounds **8** and **10**, respectively (Scheme 1).

Thiazolylhydrazones **13**–**18 **were synthesized through the Hantzsch reaction [25] of bromomethyl ketones with 2-(5-methoxy-2-oxoindolin-3-ylidene)hydrazinecarbothioamide **12** [26] (Scheme 2).

Hydrazones **19**, **21**, and **22** were prepared through condensation of the corresponding carbaldehydes and hydrazines, while compound **23** was synthesized by condensation of 5-methoxyisatin with 3-chlorobenzohydrazide. The 3-arylidenehydrazono-5-methoxyindolin-2-one derivatives **21** and **22** required the preparation of 5-methoxyisatin-3-hydrazone **20** [27], which was then reacted with 4-isopropyl or 3-chlorobenzaldehyde, respectively (Scheme 3).

The alkylation of the indole and/or hydrazone nitrogens was accomplished through different methods, starting from commercial 5-methoxyisatin or the previously reported compound **1** [20] (Scheme 4), which provided a series of *N*^1^-alkyl-substituted derivatives **24**, **26**, **28**, **32**–**34**.

Finally, compounds **36**–**39** were synthesized by condensation of 4-isopropylphenylhydrazine hydrochloride with isatins **35a** [28] and **35d** [29] (Scheme 5), previously prepared through the Sandmeyer reaction [30], and commercial isatins **35b** and **35c**. Synthesis and characterization of compounds **15** [31], **25** [32] and **29** [33] have been already reported.

### 2.2. Inhibition of Amyloid Aggregation

In vitro inhibition of Aβ aggregation was assessed through a ThT fluorescence-based method [18], with the use of 2% 1,1,1,3,3,3-hexafluoroisopropanol (HFIP) as aggregation enhancer. In this medium-throughput assay, we preferred using Aβ_40_ peptide, being more manageable than Aβ_42 _and less prone to the formation of preaggregates [18]. Samples of Aβ were co-incubated with test molecules in PBS at 100 µM concentration and the antiaggregating activities were measured after 2 h of incubation at 25 °C. For compounds showing >80% Aβ_40_ aggregation inhibition, IC_50_s were determined.

The already reported antifibrillogenic activity of the indolylhydrazones [19,20] allowed us to identify (3*Z*)-5-methoxy-1*H*-indole-2,3-dione 3-[(4-isopropylphenyl)hydrazone] (**1**, Figure 1) as a hit compound, displaying strong inhibition of Aβ_40 _aggregation (IC_50_ 0.43 µM) [20]. Compound **1** proved to be quite stable at pH 7.4 and 37 °C (half-life 24 h) and to inhibit AChE (IC_50_ 6.25 µM) [22].

An early objective of the present study was to replace the 4-*i*Pr substituent with phenyl groups bearing polar and apolar electron-withdrawing substituents (compounds **4**–**6**, Table 1). Such modifications determined a drop of the activity, and only the 3′,5′-bis(trifluoromethyl) derivative **6** showed IC_50_ in the low micromolar range (9.9 µM), which resulted however 25-fold less potent than compound **1**.

The replacement of the hydrazone phenyl ring with a number of other aromatic or heteroaromatic rings was carried out for investigating the effects of bulkiness and/or additional π-π interactions on the anti-aggregating potency. The bioisosteric replacement of the phenyl group with pyrid-2-yl (**7**) resulted in a much lower activity (IC_50_ 90 μM), whereas the 1-naphthyl moiety in compound **8** did recover a fair potency (IC_50_ 28 µM), indicating that additional aromatic interactions may improve the antiaggregating potency. In contrast, quinolyl analogues **9** and **10**, (similarly to the pyrid-2-yl congener **7**), showed lower potency, and the 7-chloroquinolin-4-yl derivative **11** resulted a very weak inhibitor.

Further information on the bioisosteric replacement of phenylhydrazone ring was achieved with thiazolylhydrazones **13**–**18** (Table 2). An exploration of the substituents on the thiazole moiety clearly indicated a preference for 4-phenylthiazole derivative **15**, which indeed proved to be a potent inhibitor of Aβ aggregation (IC_50_ 1.2 µM), apparently due to additional hydrophobic/aromatic interactions attained by the phenyl group at the C4 position of the thiazole ring.

The 3′-substituted congeners of 4-phenylthiazol-2-yl derivatives **16**–**18**, regardless the physicochemical feature of the *meta* substituent, retained antiaggregating activity in the micromolar range, but resulted 8-to-30-fold less potent than **15**, thereby suggesting critical steric requirements for these derivatives. In contrast, smaller alkyl substituents, namely methyl or chloromethyl in compounds **13** and **14**, respectively, displayed contrasting effects, with the 4-chloromethyl derivative **13** retaining a fair anti-aggregating potency (IC_50_ 13 µM), and 4,5-dimethyl analogue resulting a very weak inhibitor.

According to our investigation strategy (Figure 2), the following step was aimed at exploring the effects on the inhibition of Aβ aggregation of a few variations of the linker (length and the chemical nature) between the two structural moieties (Table 3).

A comparison of the data in Table 3 with that of the hit compound **1** revealed a 100-fold decrease of potency (from 0.43 to 41 µM), when linker was changed in the 1*H*-indole derivative **19**, likely due to the lack of intramolecular hydrogen bond (IMHB) between the hydrazone NH and the carbonyl O at position 2 of isatin, which favors co-planarity between the two aromatic moieties. The inhibitory activity was maintained in the low micromolar range by the aza-derivatives **21** (IC_50_ 6.7 µM), that is a strict analog of **1**, and **22** (IC_50_ 11 µM), that is the 3-chloro congener of **21**, whereas a sharp drop of activity was observed for the carboxyhydrazide derivative **23**. The antiaggregating potency of compounds **21**–**23** appears to be related to their lipophilicity, as assessed by logP values calculated with the ACDLab software (4.97, 4.43 and 3.53 for **21**, **22** and **23**, respectively).

The introduction of alkyl and phenylalkyl groups at the indole N and methylation of the hydrazone NH in the hit structure **1** were then investigated (Table 4). Inhibition data highlighted that small and bulky alkyls on the 1*H*-indole nitrogen of **1** were tolerated, so that the *N*-methyl (**26**), *N*-cyclopropyl (**28**) and *N*-phenylalkyl (**32**–**34**) derivatives resulted almost equipotent with the hit compound **1** in the submicromolar range (IC_50_s ranging from 0.53 to 0.83 µM). A nonlinear relation between the antifibrillogenic potency and lipophilicity of the *N*-alkyl groups exists for these compounds, the *N*-benzyl derivative **32** being the most potent one within the subset (IC_50_ 0.53 µM). In contrast, methylation of the hydrazone nitrogen (**24**) resulted in a more than 10-fold decrease in the inhibition potency compared with the respective non-methylated compound (**26**), which proves that *N*-methylation of hydrazone N may prevent IMHB formation with carbonyl O at 2-position [22] and/or may introduce a steric effect that hinders hydrazone NH from acting as HB-donor (HBD) in the interaction with the Aβ peptide.

Finally, compounds **36**–**39**, bearing diverse lipophilic and hydrophilic substituents on the indole ring (Table 5), were tested for the effects on Aβ aggregation. The introduction of *n*-butyl (**36**) or Br (**37**) at 5- and 7-position, respectively, resulted detrimental, leading in the latter case to complete loss of activity. The evidence of an enhancing role of hydrophilic substituents (particularly 5,6-dihydroxy derivatives) for an efficient inhibition of Aβ_40_ aggregation, emerging from our previous works [20,21], was endeavored by introducing a net negative charge with the sulfonate salt **38** and a protecting group for catechol derivatives, i.e., the 5,6-methylenedioxy substituent, in **39**. Both modifications resulted poorly effective, and the two compounds showed inhibitory potencies much lower than that of compound **1**.

To place on a more quantitative basis the above SAR trends, the lipophilicity of a number of isatin arylhydrazone derivatives was measured by an RP-HPLC method [34,35]. The polycratic capacity factors (log *k’*_w_) were determined (details in the Experimental Section). Lipophilicity descriptors (i.e., 1-octanol–water partition coefficients) were also calculated (CLOGPs) using two computational tools (Bio-Loom software, v. 1.7 and ACDLabs software, release 10.0). The ACDLabs software was used also for calculating descriptors of polarizability and size, namely molar refractivity (CMR) and molar volume (MV). The experimental lipophilicity parameters (log *k*’_w_), along with CMR and MV, are reported in Table 6 for 18 compounds achieving finite IC_50_s (<100 µM) in the Aβ aggregation assay.

Log *k’*_w_ values resulted reasonably correlated, over a range of about five log units, with CLOGPs from ACDLabs software (*r*^2^ = 0.805; Supplementary Materials Figure S1). The linear equation correlating log *k’*_w_ and CLOGP has a slope close to one (+1.03) and an intercept of about +0.70, the latter revealing a systematic positive deviation from linearity, likely due to the so-called “silanophilic” interactions on the silica-based C18 stationary phase [36,37,38].

**Figure 3:**
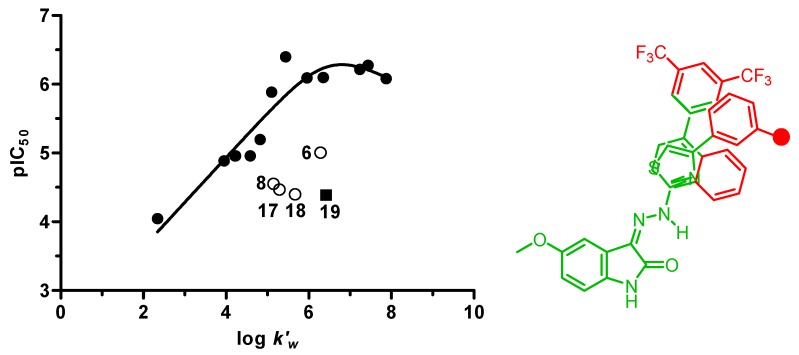
**Left**: Bilinear relationship (Kubinyi’s model) between Aβ aggregation inhibition potency (pIC_50_) and lipophilicity, as assessed by RP-HPLC (log *k’*_w_). Data points deviating from the bilinear relationship are represented as unfilled circles (**6**, **8**, **17**, **18**) and filled square (the only non-isatin derivative, (**19**). **Right**: Superposition of bidimensional structures of molecules **6**, **8**, **17** and **18** behaving as strong outliers; moieties that disrupt binding to Aβ peptides, most likely because of steric effects (ligands’ exceeding volumes), are colored in red.

A biphasic relationship was observed for the majority of examined (thirteen out of eighteen) compounds between pIC_50_ and log *k*’_w_ (Figure 3).

Omitting from the regression analysis the strong outliers **6**, **8**, and **17**–**19**, the following bilinear Equation (1) was obtained according to the Kubinyi’s model [39]:

pIC_50_ = 0.65 (±0.11) log *k’*_w_ − 0.95 (±0.33) log (β*k’*_w_ + 1) + 2.32 n = 13, *r*^2^ = 0.900, *s* = 0.271, logβ = −6.47, optimum log *k’*_w_ = 6.80 (1)

In the above equation, β is the nonlinear term of the Kubinyi’s equation; n represents the number of data points, *r*^2^ the coefficient of determination (squared correlation coefficient), and s the standard deviation of the regression equation; 95% confidence intervals of the regression coefficients are given in parentheses. The optimum value of log *k’*_w_, as calculated from the above equation, is 6.80. Similar, but statistically slightly poorer, bilinear correlation was obtained with CMR (*r*^2^ = 0.704; Supplementary Materials Figure S3).

The pIC_50_ values of the outliers **6**, **8**, **17**–**19** resulted 1.2–1.5 log units lower than those expected by the bilinear relationship with lipophilicity. These deviations may be reasonably explained as follows: (i) compound **19** is the only phenylhydrazone of 1*H*-indole-3-carbaldehyde, and not of isatin, whose carbonyl O at position 2 may play a critical role either in forming IMHB with the hydrazone NH or in interacting with counterparts of the amyloid peptides through dipole-dipole interactions or HBs; (ii) compounds **6** and **8** (as well as the quinoline-bearing compounds **9**–**11**), **17** and **18**, regardless of lipophilicity, may undergo detrimental steric effects when approaching and interacting into the binding site of Aβ peptide because of bulky moieties around the phenylhydrazone (Figure 3). As for the 4-phenylthiazol-2-yl-containing derivatives, compounds **17** and **18**, bearing OMe or Br in *meta* position of phenyl, resulted less active than the respective unsubstituted analog **15** and the less bulky 3′-OH congener **16**, which in fact fit the bilinear equation model.

Taken together, our data suggested that both the 5-methoxy substituent on the indolin-2-one moiety and the 4-isopropyl substituent on the phenylhydrazone moiety play an essential role in maintaining the Aβ_40_ antiaggregating potency in the submicromolar range. *N*-methylation of hydrazone NH, elimination of 2-carbonyl, introduction of other substituents at positions 5, 6 and 7 of the indolin-2-one nucleus and replacement of the isopropyl group with (hetero)aromatic moieties proved unfavorable, whereas alkylation of the indole nitrogen was tolerated at least up to an optimal lipophilicity corresponding, in our case, to the *N*^1^-benzyl derivative **32**.

Based on data in Table 1, Table 2, Table 3, Table 4 and Table 5, compound **28** was selected for further biological and biophysical studies, aimed at elucidating the mechanism of disruption of Aβ aggregation, and evaluating the activity in biochemical assays related to neurodegeneration, and cytoprotection in a cell model of Aβ toxicity. Starting from the evidence that a bulky substituent led to a net improvement of antiaggregating activity (Table 5), we chose the *N*^1^-cyclopropyl derivative **28** as the best compromise between in vitro activity and aqueous solubility. As expected, solubility of **28** resulted lower than lead compound **1** (21 vs. 71 µM [22] in Tris/HCl buffer, pH 7.4 at 25 °C), but sufficient to allow performing the co-incubation assays where 20 µM represents the upper concentration threshold to test.

### 2.3. Kinetics of Aβ_42_ Fibrillization

**Figure 4:**
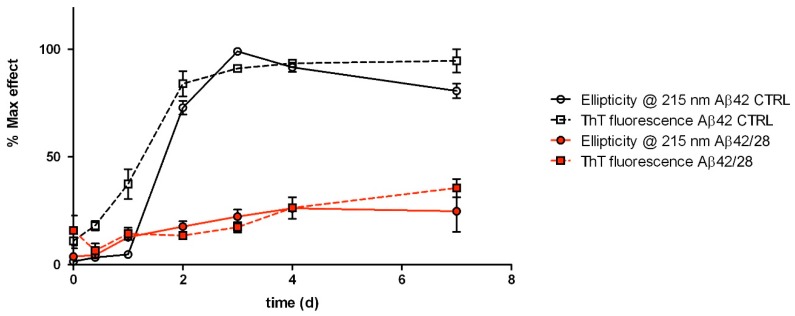
Time-course aggregation kinetics of 50 µM Aβ_42_ control (black lines) and Aβ_42_ with 20 µM **28** (red lines). Results are expressed as percentage of the maximal values of CD ellipticity at 215 nm (circles, full lines) and ThT fluorescence (squares, dotted lines) of control peptide sample.

For ThT and circular dichroism (CD) time-course measures of Aβ aggregation (50 µM), alone or in the presence of **28** (20 µM), samples were prepared in PBS containing 2% *v*/*v* ethanol as the cosolvent, and incubated at 37 °C. Aβ_42_ peptide was preferred, being the principal responsible of amyloid burden in AD brain. Kinetics of amyloid aggregation of Aβ_42_ was followed by means of ThT fluorescence and CD absorption. In the latter case, we monitored the increase of the negative band at 215 nm, probing the random coil to β-sheet transition in folding peptide. Results depicted in Figure 4 show a fast fibrillization of self-aggregating peptide (dotted black line), with fibrils already detectable in large amount in the first 48 h of incubation. Soluble β-rich species are in turn massively detected after two days (full black line), suggesting that the fibrillization process takes place by quickly recruiting oligomer intermediates. On the other hand, samples co-incubated with **28** showed a sharply reduced amount either of β-sheet arranged and ThT-stained aggregates (red lines), reaching only after one week a fibril content of about 30% compared with that of control peptide.

### 2.4. Protection Assays Against Oxidative and Cytotoxic Effects

Protection from Aβ_42_-induced cytotoxicity was measured for compound **28** in a conventional cell-based assay, measuring cell viability by means of MTT reduction [40]. Antioxidant activity of **28** was assessed in an H_2_O_2_-induced oxidation cell model. Reactive oxygen species (ROS) production was detected by means of a spectrofluorometric measure of the fluorescent probe 2′,7′-dichlorofluorescein (DCF), formed by oxidation of 2′,7′-dichlorodihydrofluorescein (DCFH) [41]. In both assays, human SH-SY5Y neuroblastoma cell line was used.

**Figure 5:**
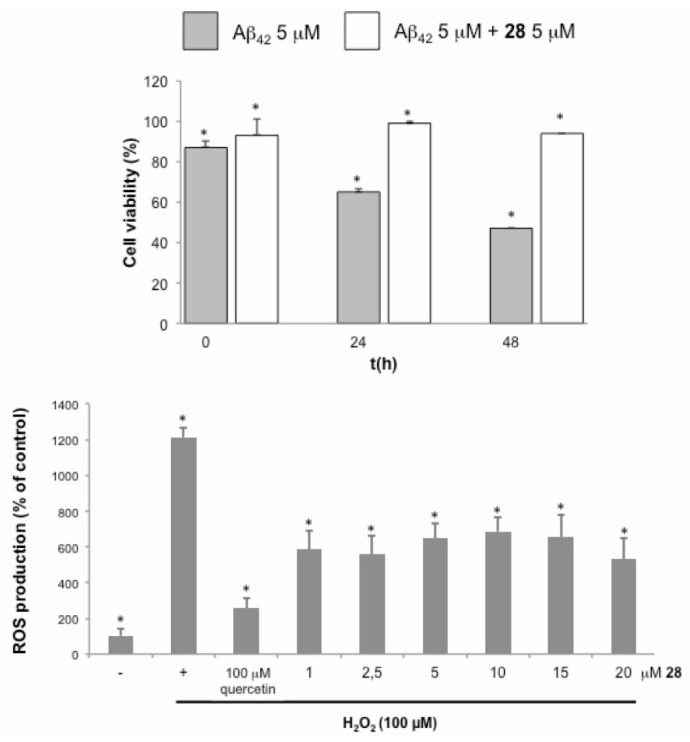
Cytoprotection of SH-SY5Y cells by compound **28**. Top, viability of cells at 0, 24 and 48 h incubated with 5 µM Aβ_42_ alone (grey bars) or in coincubation with equimolar **28** (white bars). The percentage of MTT reduction is relative to control cells. Bottom, radical scavenging activity of increasing concentrations of **28** (0 to 20 µM) in the presence of 100 µM hydrogen peroxide (DCFH-DA assay; control (−): non-treated cells; (+): positive control). Values are expressed as mean ± S.E.M. from six replicates, significantly different from control (untreated cells) as estimated by the Student’s *t* test (* *p* < 0.01).

The aggregates formed by 5 µM Aβ_42_ in cultured SH-SY5Y cells produced around 50% of cell death within two days, while cells co-incubated with equimolar 5 µM Aβ_42_ and **28** were fully viable in the same time frame (Figure 5, top). This result agreed with the observed activity in vitro and confirmed that previously observed in a close set of congeners [21,22]. Furthermore, to better investigate the cytoprotection exerted by this class of compounds, the antioxidant activity of compound **28** was investigated. Figure 5 (bottom) shows the radical-scavenging effects of increasing concentrations (0 to 20 µM) of **28** against oxidation induced by 100 µM hydrogen peroxide. Quercetin, a well-known natural antioxidant, was used as reference compound. ROS scavenging was exerted by **28** even at lower concentrations and reached a maximum effect at 20 µM concentration, which is about 50% of the effect shown by 100 µM quercetin. Thus, the antioxidant activity of **28** is supposed to be comparable to that of quercetin in the assay conditions herein used.

## 3. Materials and Methods

### 3.1. Chemistry

Commercial reagents and solvents were purchased from Sigma-Aldrich (Milan, Italy). Melting points (mp) were determined by the capillary method on a Stuart SMP3 electrothermal apparatus (Bibby Scientific, Milan, Italy). IR spectra were recorded using potassium bromide disks on a Spectrum One FT-IR spectrophotometer (Perkin Elmer, Milan, Italy); only the most significant IR absorption bands are reported. ^1^H-NMR spectra were recorded in DMSO-*d*_6_ on a Mercury 300 spectrometer (Varian, Cernusco sul Naviglio, Italy). Chemical shifts are expressed in δ (ppm) and the coupling constants *J* in Hz. The following abbreviations were used: s, singlet; d, doublet; t, triplet; qn, quintuplet; sx, sextuplet; ep, septuplet; dd, double doublet; m, multiplet; br s, broad singlet. Chromatographic separations were performed on silica gel 63–200 (Merck, Milan, Italy). ESI-MS was performed with an electrospray interface and an ion trap mass spectrometer (1100 Series LC/MSD Trap System, Agilent, Palo Alto, CA, USA). The sample was infused via a KD Scientific syringe pump at a rate of 10 mL/min. The pressure of the nebulizer gas was 15 psi. The drying gas was heated to 350 °C at a flow of 5 L/min. Full-scan mass spectra were recorded in the mass/charge (*m*/*z*) range of 50–800 amu. For some representative compounds, HRMS experiments were performed with a dual electrospray interface (ESI) and a quadrupole time-of-flight mass spectrometer (Q-TOF, Agilent 6530 Series Accurate-Mass Quadrupole Time-of-Flight LC/MS, Agilent Technologies Italia S.p.A., Cernusco sul Naviglio, Italy). The ^1^H-NMR and HPLC purities of all the newly synthesized final products were always higher than 95%. Compounds **2** [24], **12** [26], **15** [31], **20** [27], **25** [32], **29** [33], **35a** [28] and **35d** [29] have been prepared according to quoted references. Their analytical data agreed with those reported in the literature.

#### 3.1.1. Synthesis of 5-methoxy-1H-indol-2,3-dione 3-arylhydrazones **4**–**7**, **9** and **11**

5-Methoxyisatin (1 mmol) was dissolved in methanol (5 mL) with gentle heating, then arylhydrazine (2.4 mmol) was slowly added with stirring at room temperature. After stirring overnight, the resulting precipitate was filtered and crystallized or the reaction mixture was evaporated to dryness and the residue suitably crystallized.

*(Z)-4-(2-(5-Methoxy-2-oxoindolin-3-ylidene)hydrazinyl)benzonitrile* (**4**). Yield: 60%. ^1^H-NMR δ 3.76 (s, 3H, 5-OCH_3_), 6.83–6.85 (m, 2H, H-6 and H-7), 7.44 (d, 1H, *J* = 2.2 Hz, H-4), 7.60 (d, 2H, *J* = 8.8 Hz, H-3′ and H-5′), 7.78 (d, 2H, *J* = 8.8 Hz, H-2′ and H-6′), 10.92 (s, 1H, NH), 12.77 (s, 1H, NNH). ESI-MS *m*/*z* = 290.8 (89%) [M − H]^−^. IR (KBr): 3359, 3218, 2217, 1686, 1606, 1571, 1515, 1488, 1151 cm^−1^. Mp 246–249 °C from isopropanol.

*(Z)-5-Methoxy-3-(2-(4-(trifluoromethyl)phenyl)hydrazono)indolin-2-one* (**5**). Yield: 50%. ^1^H-NMR δ 3.76 (s, 3H, 5-OCH_3_), 6.80–6.87 (m, 2H, H-6 and H-7), 7.15 (d, 1H, *J* = 1.1 Hz, H-4), 7.60 (d, 2H, *J* = 8.5 Hz, H-2′ and H-6′), 7.68 (d, 2H, *J* = 8.5 Hz, H-3′ and H-5′), 10.90 (s, 1H, NH), 12.80 (s, 1H, NNH). ESI-MS *m*/*z* = 333.8 (100%) [M − H]^−^. IR (KBr): 3166, 1684, 1563, 1487, 12887, 1161 cm^−1^. Mp 228–230 °C from methanol.

*(Z)-3-(2-(3,5-bis(trifluoromethyl)phenyl)hydrazono)-5-methoxyindolin-2-one* (**6**). Yield: 84%. ^1^H-NMR δ 3.77 (s, 3H, 5-OCH_3_), 6.82 (d, 1H, *J* = 8.4 Hz, H-7), 6.87 (dd, 1H, *J* = 8.4 Hz, *J* = 2.6 Hz, H-6), 7.24 (d, 1H, *J* = 2.4 Hz, H-4), 7.61 (s, 1H, H-4′), 8.12–8.14 (m, 2H, H-2′ and H-6′), 10.89 (s, 1H, NH), 12.86 (s, 1H, NNH). ESI-MS *m*/*z* = 401.8 (100%) [M − H]^−^. HRMS (ESI) *m*/*z* [M − H]^−^ calcd for C_17_H_10_F_6_N_3_O_2_^−^ 402.0683; found, 402.0685. IR (KBr): 3164, 1684, 1564, 1487, 1374, 1279, 1163 cm^−1^. Mp 240–242 °C (dec.) from methanol.

*(Z)-5-Methoxy-3-(2-(pyridin-2-yl)hydrazono)indolin-2-one* (**7**). Yield: 80%. ^1^H-NMR δ 3.78 (s, 3H, 5-OCH_3_), 6.76 (d, 1H, *J* = 8.5 Hz, H-7), 6.87 (d, 1H, *J* = 8.5 Hz, H-6), 7.02 (s, 1H, H-4), 7.43–7.45 (m, 1H, H-4′), 7.77–7.80 (m, 2H, H-5′, H-6′), 8.27 (br s, 1H, H-3′), 10.41 (s, 1H, NH), 11.07 (s, 1H, NNH). ESI-MS, *m*/*z* = 266.8 (100%) [M − H]^−^, 291.0 (100%) [M + Na]^+^. IR (KBr): 1715, 1691, 1570, 1484, 1269, 1197 cm^−1^. Mp > 250 °C from methanol.

*(Z)-5-Methoxy-3-(2-(quinolin-2-yl)hydrazono)indolin-2-one* (**9**). Yield: 94%. ^1^H-NMR δ 3.76 (s, 3H, 5-OCH_3_), 6.73 (d, 1H, *J* = 8.4, H-7), 6.80–6.88 (m, 1H, H-6), 7.23–7.29 (m, 1H, H-4), 7.44–7.76 (m, 4H, H-5′, H-6′, H-7′and H-8′), 7.80–8.10 (m, 2H, H-3′ and H-4′), 10.24 (s, 1H, NH), 12.00 (br s, 1H, NNH). ESI-MS *m*/*z* = 316.8 (100%) [M − H]^−^, 341.0 (100%) [M + Na]^+^. IR (KBr): 3430, 2919, 1688, 1634, 1476, 1302, 1186 cm^−1^. Mp 227–234 °C from ethanol.

*(Z)-3-(2-(7-Chloroquinolin-4-yl)hydrazono)-5-methoxy-indolin-2-one* (**11**). Yield: 82%. ^1^H-NMR δ 3.79 (s, 3H, 5-OCH_3_), 6.60–6.64 (m, 1H, H-7), 6.80–6.90 (m, 2H, H-4 and H-6), 7.25–7.26 (m, 1H, H-5′), 7.68–7.76 (m, 2H, H-2′ and H-3′), 8.03–8.12 (m, 1H, H-6′), 8.30 (s, 1H, H-8′), 9.67 (br s, 1H, NH), 11.20 (br s, 1H, NNH). ESI-MS *m*/*z* = 350.8 (100%) [M − H]^−^, 352.8 (34%) [M − H]^−^ + 2, 353.0 (39%) [M + H]^+^, 355.0 (14%) [M + H]^+^ + 2. IR (KBr): 3403, 3166, 1703, 1630, 1381, 1185 cm^−1^. Mp > 250 °C from ethanol.

#### 3.1.2. Synthesis of 3-arylhydrazono-indolin-2-ones **8** and **10**

Compounds **8** and **10** were prepared from azo coupling of aryldiazonium salts **3a**,**b** with 5-methoxyindolin-2-one **2**, previously described [24]. Aryldiazonium salts were prepared starting to corresponding amines (21.5 mmol) in HCl 6N (11 mL) with water solution (7.5 mL) of sodium nitrite (23 mmol). This solution was added to solution of indol-2-one (21.5 mmol) and sodium acetate (43 mmol) in methanol (59 mL) at 0 °C. The resulting precipitate was filtered and purified.

*(Z)-5-Methoxy-3-(2-(naphthalen-1-yl)hydrazono)indolin-2-one* (**8**). Yield: 40%. ^1^H-NMR δ 3.78 (s, 3H, 5-OCH_3_), 6.86 (s, 2H, H-6 and H-7), 7.20 (s, 1H, H-4), 7.53–7.68 (m, 4H, H-4′, H-5′, H-6′ and H-7′), 7.83–7.89 (m, 2H, H-2′ and H-3′), 7.97 (d, 1H, *J* = 8.1 Hz, H-8′), 11.04 (s, 1H, NH), 13.83 (s, 1H, NNH). ESI-MS *m*/*z* = 315.8 (100%) [M − H]^−^. IR (KBr): 3014, 1672, 1562, 1484, 1194, 1156 cm^−1^. Mp > 250 °C from hexane/ethyl acetate 70:30 (*v*/*v*).

*(Z)-5-Methoxy-3-(2-(quinolin-8-yl)hydrazono)indolin-2-one* (**10**). Yield: 50%. ^1^H-NMR δ 3.78 (s, 3H, 5-OCH_3_), 6.84–6.85 (m, 2H, H-6 and H-7), 7.21–7.22 (m, 1H, H-4), 7.61–7.67 (m, 3H, H-3′, H-4′ and H-6′), 7.96 (d, 1H, *J* = 7.0 Hz, H-5′), 8.39 (d, 1H, *J* = 8.4 Hz, H-2′), 8.91–8.93 (m, 1H, H-7′), 10.88 (s, 1H, NH), 13.92 (s, 1H, NNH). ESI-MS *m*/*z* = 341.0 (64%) [M + Na]^+^, 316.8 [M − H]^–^. IR (KBr): 3435, 1675, 1649, 1522, 1194 cm^−1^. Mp > 250 °C from dichloromethane/methanol 95:05 (*v*/*v*).

#### 3.1.3. Synthesis of 5-Methoxy-3-(2-thiazolylhydrazono)indolin-2-ones **13**–**18**

Compounds **13**–**18** were prepared adding to a solution of **12** [26] (1 mmol) in ethanol (36 mL) 1 mmol of appropriate bromomethylketone (1-bromo-3-chloropropan-2-one for **13**; 3-bromo-2-butanone for **14**; α-bromoacetophenone for **15**; 3-hydroxy-α-bromoacetophenone for **16**; 3-methoxy-α-bromoacetophenone for **17**; 3,α-dibromoacetophenone for **18**) at room temperature. After stirring overnight the resulting precipitate was filtered and purified by column chromatography or crystallization.

*(Z)-3-(2-(4-(Chloromethyl)thiazol-2-yl)hydrazono)-5-methoxyindolin-2-one* (**13**). Yield: 40%. ^1^H-NMR δ 3.76 (s, 3H, 5-OCH_3_), 4.67 (s, 2H, CH_2_Cl). 6.86 (d, 1H, *J* = 8.4 Hz, H-7), 6.91 (dd, 1H, *J* = 8.4 Hz, *J* = 2.2 Hz, H-6), 7.04 (d, 1H, *J* = 2.2 Hz, H-4), 7.25 (s, 1H, H-5′), 11.05 (s, 1H, NH), 13.33 (s, 1H, NNH). ESI-MS *m*/*z* = 320.7 (100%) [M − H]^−^, 322.7 (48%) [M − H]^−^ + 2. IR (KBr): 3436, 2946, 1683, 1556, 1476, 1168 cm^−1^. Mp 153–155 °C (dec.) from chloroform/methanol 96:04 (*v*/*v*).

*(Z)-3-(2-(4,5-Dimethylthiazol-2-yl)hydrazono)-5-methoxyindolin-2-one* (**14**). Yield: 40%. ^1^H-NMR δ 2.12 (s, 3H, CH_3_), 2.25 (s, 3H, CH_3_), 3.76 (s, 3H, 5-OCH_3_), 6.85 (d, 1H, *J* = 8.4 Hz, H-7), 6.89 (dd, 1H, *J* = 8.4 Hz, *J* = 2.2 Hz, H-6), 7.00 (d, 1H, *J* = 2.2 Hz, H-4), 10.98 (s, 1H, NH), 13.19 (s, 1H, NNH). ESI-MS *m*/*z* = 300.8 (100%) [M − H]^−^, 325.0 (94%) [M + Na]^+^. IR (KBr): 3446, 1686, 1544, 1486, 1162 cm^−1^. Mp 160–164 °C from ethanol.

*(Z)-3-(2-(4-(3-Hydroxyphenyl)thiazol-2-yl)hydrazono)-5-methoxyindolin-2-one* (**16**). Yield: 44%. ^1^H-NMR δ 3.84 (s, 3H, 5-OCH_3_), 6.69–6.72 (m, 1H, H-4″), 6.86 (d, 1H, *J* = 8.4 Hz, H-7), 6.91 (dd, 1H, *J* = 8.4 Hz, *J* = 2.4 Hz, H-6), 7.06 (d, 1H, *J* = 2.4 Hz, H-4), 7.19 (t, 1H, *J* = 8.1 Hz, H-5″), 7.29–7.31 (m, 2H, H-2″ and H-6″) 7.53 (s, 1H, H-5′), 9.47 (s, 1H, 3″-OH), 11.06 (s, 1H, NH), 13.41 (s, 1H, NNH). ESI-MS *m*/*z* = 364.8 (100%) [M − H]^−^. HRMS (ESI) *m*/*z* [M + H]^+^ calcd for C_18_H_15_N_4_O_3_S^+^ 367.0859; found, 367.0833. IR (KBr): 3431, 1551, 1523, 1163 cm^−1^. Mp > 250 °C from ethanol.

*(Z)-5-Methoxy-3-(2-(4-(3-methoxyphenyl)thiazol-2-yl)hydrazono)indolin-2-one* (**17**). Yield: 91%. ^1^H-NMR δ 3.78 (s, 3H, 3″-OCH_3_), 3.80 (s, 3H, 5-OCH_3_), 6.87–6.93 (m, 3H, H-6, H-7 and H-4″), 7.06 (d, 1H, *J* = 2.2 Hz, H-4), 7.32 (t, 1H, *J* = 7.9 Hz, H-5″), 7.43–7.48 (m, 2H, H-6″ and H-2″), 7.66 (s, 1H, H-5′), 11.07 (s, 1H, NH), 13.39 (s, 1H, NNH). ESI-MS *m*/*z* = 378.8 (100%) [M − H]^−^. IR (KBr): 3171, 2926, 1679, 1551, 1171 cm^−1^. Mp > 250 °C from ethanol.

*(Z)-3-(2-(4-(3-Bromophenyl)thiazol-2-yl)hydrazono)-5-methoxyindolin-2-one* (**18**). Yield: 30%. ^1^H-NMR δ 3.77 (s, 3H, 5-OCH_3_), 6.87 (d, 1H, *J* = 8.4 Hz, H-7), 6.92 (dd, 1H, *J* = 2.4 Hz, *J* = 8.4 Hz, H-6), 7.06 (d, 1H, *J* = 2.4 Hz, H-4), 7.37 (t, 1H, *J* = 7.9 Hz, H-5″), 7.49–7.53 (m, 1H, H-4″), 7.78 (s, 1H, H-5′), 7.89–7.91 (m, 1H, H-6″), 8.07 (t, 1H, *J* = 1.6 Hz, H-2″), 11.07 (s, 1H, NH), 13.40 (s, 1H, NNH). ESI-MS *m*/*z* = 426.8 (99%) [M − H]^−^, 428.7 (100%) [M − H]^−^ + 2. IR (KBr): 1683, 1552, 1483, 1155, 718 cm^−1^. Mp 240–242 °C (dec.) from dichloromethane/methanol 92:8 (*v*/*v*).

#### 3.1.4. Synthesis of Compound **19**

4-isopropylphenylhydrazine hydrochloride (1.8 mmol) was added to a solution of 5-methoxy-indole-3-carbaldehyde (1 mmol) in methanol (5 mL). After stirring overnight at room temperature, the resulting precipitate was filtered and crystallized.

*(E)-3-(2(4-Isopropylphenyl)hydrazonomethyl))-5-methoxy-1H-indole* (**19**). Yield: 30%; ^1^H-NMR δ 1.23 (d, 6H, *J* = 6.7 Hz, CH(CH_3_)_2_), 2.97 (ep, 1H, *J* = 6.7 Hz, CH(CH_3_)_2_), 3.82 (s, 3H, 5-OCH_3_), 6.80 (dd, 1H, *J* = 8.8 Hz, *J* = 2.4 Hz, H-6), 6.94 (d, 2H, *J* = 8.1 Hz, H-3′ and H-5′), 7.07 (d, 2H, *J* = 8.1 Hz, H-2′ and H-6′), 7.28 (d, 1H, *J* = 8.8 Hz, H-7), 7.54 (d, 1H, *J* = 2.5 Hz, H-4), 7.78 (d, 1H, *J* = 2.5 Hz, H-2), 8.05 (s, 1H, CH=N), 9.68 (s, 1H, NH), 11.14 (s, 1H, NNH). ESI-MS *m*/*z* = 305.9 (100%) [M − H]^−^. IR (KBr): 3340, 1485, 1296, 1196 cm^−1^. Mp 175–177 °C from ethanol.

#### 3.1.5. Synthesis of 3-(Benzylidenehydrazono)-5-methoxyindolin-2-ones **21** and **22**

Compound **20** [27] (1 mmol) was dissolved in methanol (20 mL), then the suitable benzaldehyde (1 mmol) was added and the mixture was heated to reflux. The resulting precipitate was filtered and purified by column chromatography.

*(Z)-3-((E)-(4-Isopropylbenzylidene)hydrazono)-5-methoxyindolin-2-one* (**21**). Yield: 30%; ^1^H-NMR δ 1.23 (d, 6H, *J* = 6.7 Hz, CH(CH_3_)_2_), 2.97 (ep, 1H, *J* = 6.7 Hz, CH(CH_3_)_2_), 3.69 (s, 3H, 5-OCH_3_), 6.82 (d, 1H, *J* = 8.4 Hz, H-7), 7.00 (dd, 1H, *J* = 3.0 Hz, *J* = 8.4 Hz, H-6), 7.45 (d, 2H, *J* = 8.1 Hz, H-3′ and H-5′), 7.54 (d, 1H, *J* = 3.0 Hz, H-4), 7.88 (d, 2H, *J* = 8.1 Hz, H-2′ and H-6′), 8.58 (s, 1H, N=CH), 10.62 (br s, 1H, NH). ESI-MS *m*/*z* = 319.9 (61%) [M − H]^−^, 344.0 (100%) [M + Na]^+^. HRMS (ESI) *m*/*z* [M + H]^+^ calcd for C_19_H_20_N_3_O_2_^+^ 322.1537; found, 322.1550. IR (KBr): 3390, 2956, 1733, 1485, 1296, 1196 cm^−1^. Mp 223–225 °C from chloroform/methanol 95:5 (*v*/*v*).

*(Z)-3-((E)-(3-chlorobenzylidene)hydrazono)-5-methoxyindolin-2-one* (**22**). Yield: 30%. ^1^H-NMR δ 3.78 (s, 3H, 5-OCH_3_), 6.86 (d, 1H, *J* = 8.5 Hz, H-7), 6.95 (dd, 1H, *J* = 8.5 Hz, *J* = 2.2 Hz, H-6), 7.15 (d, 1H, *J* = 2.2 Hz, H-4), 7.64 (t, 1H, *J* = 7.5 Hz, H-5′), 7.70–7.75 (m 1H, H-4′), 7.80–7.87 (m, 1H, H-6′), 7.90–7.93 (m, 1H, H-2′), 8.70 (s, 1H, N=CH), 10.20 (br s, 1H, NH). ESI-MS *m*/*z* = 313.05 (100%) [M − H]^−^, 315.04 (34%) [M − H]^−^ + 2. IR (KBr): 3465, 1745, 1626, 1487 cm^−1^. Mp 230–235 °C from ethyl acetate/hexane 60:40 (*v*/*v*).

#### 3.1.6. Synthesis of Carboxyhydrazide **23**

5-Methoxyisatin (1 mmol) was dissolved in methanol (5 mL), then 3-chloro-benzohydrazide (2 mmol) was added at room temperature and, after stirring overnight, the reaction mixture was evaporated to dryness and the residue purified by column chromatography.

*3-Chloro-N’-(5-methoxy-2-oxoindolin-3-ylidene)benzohydrazide* (**23**). Yield: 40%. ^1^H-NMR δ 3.77 (s, 3H, 5-OCH_3_), 6.87 (d, 1H, *J* = 8.5 Hz, H-7), 6.97 (dd, 1H, *J* = 8.5 Hz, *J* = 2.5 Hz, H-6), 7.12 (d, 1H, *J* = 2.5 Hz, H-4), 7.64 (t, 1H, *J* = 7.8 Hz, H-5′), 7.74–7.77 (m, 1H, H-4′), 7.80–7.83 (m, 1H, H-6′), 7.87–7.89 (m, 1H, H-2′), 11.26 (br s, 1H, NH), 13.83 (br s, 1H, NNHCO). ESI-MS *m*/*z* = 351.9 (45%) [M + Na]^+^, 353.9 (15%) [M + Na]^+^ + 2. IR (KBr): 3246, 1681, 1540, 1486, 1252, 1152 cm^−1^. Mp 236–238 °C from dichloromethane/methanol 96:4 (*v*/*v*).

#### 3.1.7. Synthesis of N-alkylisatin Derivatives **24**, **30** and **31**

5-methoxyisatin (1 mmol) or compound **1** [20] were dissolved in DMSO (1.5 mL), then K_2_CO_3_ (1.5 mmol) and suitable alkyl halide (1 mmol; methyl iodide for **24**, 1-bromo-2-phenylethane for **30**, and 1-bromo-3-phenylpropane for **31**) were added at room temperature. After addition of ice, the resulting precipitate was filtered, washed with water and used for subsequently steps without further purification.

*(Z)-3-(2-(4-Isopropylphenyl)-2-methylhydrazono)-5-methoxy-1-methylindolin-2-one* (**24**). Yield: 33%. ^1^H-NMR δ 1.19 (d, 6H, *J* = 6.9 Hz, CH(CH_3_)_2_), 2.28 (ep, 1H, *J* = 6.9 Hz, CH(CH_3_)_2_), 3.17 (s, 3H, N-CH_3_), 3.76 (s, 3H, 5-OCH_3_), 3.89 (s, 3H, N-CH_3_) 6.84 (dd, 1H, *J* = 8.4 Hz, *J* = 2.5 Hz, H-6), 6.94 (d, 1H, *J* = 8.4 Hz, H-7), 7.07 (d, 1H, *J* = 2.5 Hz, H-4), 7.27 (d, 2H, *J* = 7.7 Hz, H-3′ and H-5′), 7.51 (d, 2H, *J* = 7.7 Hz, H-2′ and H-6′). ESI-MS *m*/*z* = 360.0 (100%) [M + Na]^+^. IR (KBr): 2924, 1673, 1500, 1278, 1247, 1097 cm^−1^. Mp 66–69 °C from ethyl acetate/hexane 20:80 (*v*/*v*).

*5-Methoxy-1-phenetylindolin-2,3-dione* (**30**). Yield: 60%. ^1^H-NMR δ 2.88 (t, 2H, *J* = 7.4 Hz, NCH_2_CH_2_), 3.75 (s, 3H, 5-OCH_3_), 3.85 (t, 2H, *J* = 7.4 Hz, NCH_2_CH_2_), 7.08–7.27 (m, 8H, H-4, H-6, H-7, H-2′, H-3′, H-4′, H-5′ and H-6′). ESI-MS *m*/*z* = 280.0 (30%) [M − H]^−^. IR (KBr): 3084, 2964, 1739, 1626, 1491, 1287 cm^−1^. Mp 122–127 °C.

*5-Methoxy-1-(3-phenylpropyl)indolin-2,3-dione* (**31**). Yield: 60%. ^1^H-NMR δ 1.87 (qn, 2H, *J* = 7.7 Hz, NCH_2_CH_2_CH_2_), 2.64 (t, 2H, *J* = 7.7 Hz, NCH_2_CH_2_CH_2_), 3.66 (t, 2H, *J* = 7.7 Hz, NCH_2_CH_2_CH_2_), 3.75 (s, 3H, 5-OCH_3_), 7.06–7.27 (m, 8H, H-4, H-6, H-7, H-2′, H-3′, H-4′, H-5′ and H-6′). ESI-MS *m*/*z* = 318.0 (100%) [M + Na]^+^. IR (KBr): 2926, 1722, 1596, 1491 cm^−1^. Mp 130–137 °C.

#### 3.1.8. Synthesis of N-cyclopropyl-5-methoxyindolin-2,3-dione (**27**)

5-Methoxyisatin (2 mmol), cyclopropylboronic acid (4 mmol) and sodium carbonate (4 mmol) were mixed in dichloroethane (3 mL). Then a solution of copper(II) acetate (2 mmol) and bipyridine (2 mmol) in dichloroethane (6.6 mL) at 50 °C was added and the mixture was heated for 4 h. After cooling, a solution of ammonium chloride was added and the resulting aqueous phase was extracted with dichloromethane. *1-Cyclopropyl-5-methoxyndolin-2,3-dione* 27. Yield: 60%. ^1^H-NMR δ 0.76–0.85 (m, 2H, CH_2_ cyclopr.), 0.94–1.04 (m, 2H, CH_2_ cyclopr.), 2.66 (qn, 1H, *J* = 3.4 Hz, CH cyclopr.), 3.75 (s, 3H, 5-OCH_3_), 7.09 (d, 1H, *J* = 2.6 Hz, H-4), 7.14 (d, 1H, *J* = 8.8 Hz, H-7), 7.26 (dd, 1H, *J* = 8.8 Hz, *J* = 2.6 Hz, H-6). ESI-MS *m*/*z* = 240.0 (100%) [M + Na]^+^. IR (KBr): 2962, 2924, 1722, 1490, 1281 cm^−1^. Mp 121–123 °C.

#### 3.1.9. Synthesis of N-alkyl-3-phenylhyhydrazonoindolin-2-ones **26**, **28**, **32**–**34**

The suitable N-alkylisatins **25** [32], **27**, and **29**–**31** (1 mmol) were dissolved in methanol (5 mL), and 4-isopropylphenylidrazine hydrochloride (2 mmol) was then added. After stirring overnight, the resulting precipitate was filtered and crystallized or the reaction mixture was evaporated to dryness and the residue purified by column chromatography.

*(Z)-3-(2-(4-Isopropylphenyl)hydrazono)-5-methoxy-1-methylindolin-2-one* (**26**). Yield: 30%. ^1^H-NMR δ 1.18 (d, 6H, *J* = 7.0 Hz, CH(CH_3_)_2_), 2.86 (ep, 1H, *J* = 7.0 Hz, CH(CH_3_)_2_), 3.21 (s, 3H, NCH_3_), 3.77 (s, 3H, 5-OCH_3_), 6.88 (dd, 1H, *J* = 8.5 Hz, *J* = 2.5 Hz, H-6), 7.01 (d, 1H, *J* = 8.5 Hz, H-7), 7.14 (d, 1H, *J* = 2.5 Hz, H-4), 7.23 (d, 2H, *J* = 8.5 Hz, H-3′and H-5′), 7.36 (d, 2H, *J* = 8.5 Hz, H-2′and H-6′), 12.74 (s, 1H, NNH). ESI-MS *m*/*z* = 321.9 (100%) [M − H]^−^. IR (KBr): 2952, 2872, 1683, 1487, 1246, 1223 cm^−1^. Mp 101–103 °C from ethanol.

*(Z)-1-Cyclopropyl-3-(2-(4-isopropylphenyl)hydrazono)-5-methoxyindolin-2-one* (**28**). Yield: 50%. ^1^H-NMR δ 0.84–0.86 (m, 2H, CH_2_ cyclopr.), 1.00–1.02 (m, 2H, CH_2_ cyclopr.), 1.18 (d, 6H, *J* = 7.0 Hz, CH(CH_3_)_2_), 2.77 (qn, 1H, *J* = 3.6 Hz, CH cyclopr.), 2.85 (ep, 1H, *J* = 7.0 Hz, CH(CH_3_)_2_), 3.77 (s, 3H, 5-OCH_3_), 6.89 (dd, 1H, *J* = 8.6 Hz, *J* = 2.4 Hz, H-6), 7.09 (d, 1H, *J* = 8.6 Hz, H-7), 7.12 (d, 1H, *J* = 2.4 Hz, H-4), 7.23 (d, 2H, *J* = 8.6 Hz, H-3′e H-5′), 7.35 (d, 2H, *J* = 8.4 Hz, H-2′and H-6′), 12.74 (s, 1H, NNH). ESI-MS *m*/*z* = 347.9 (100%) [M − H]^−^, 372.1 (82%) [M + Na]^+^. IR (KBr): 3436, 1668, 1553, 1480, 1248, 1031 cm^−1^. Mp 106–109 °C from ethanol.

*(Z)-1-Benzyl-3-(2-(4-isopropylphenyl)hydrazono)-5-methoxyindolin-2-one* (**32**). Yield: 80%. ^1^H-NMR δ 1.19 (d, 6H, *J* = 7.0 Hz, CH(CH_3_)_2_), 2.86 (ep, 1H, *J* = 7.0 Hz, CH(CH_3_)_2_), 3.75 (s, 3H, 5-OCH_3_), 4.97 (s, 2H, CH_2_-Ph), 6.80 (dd, 1H, *J* = 8.6 Hz, *J* = 2.6 Hz, H-6), 6.93 (d, 1H, *J* = 8.6 Hz, H-7), 7.16 (d, 1H, *J* = 2.6 Hz, H-4), 7.25 (d, 2H, *J* = 8.6 Hz, H-3′ e H-5′), 7.31–7.33 (m, 5H, H-2″, H-3″, H-4″, H-5″ and H-6″), 7.40 (d, 2H, *J* = 8.6 Hz, H-2′and H-6′), 12.74 (s, 1H, NNH). ESI-MS *m*/*z* = 397.9 (100%) [M − H]^−^, 422.1 (34%) [M + Na]^+^. HRMS (ESI) *m*/*z* [M + H]^+^ calcd for C_25_H_26_N_3_O_2_^+^ 400.2020; found, 400.2028. IR (KBr): 3436, 2964, 1676, 1487, 1162 cm^−1^. Mp 105–109 °C from ethanol.

*(Z)-3-(2-(4-Isopropylphenyl)hydrazono)-5-methoxy-1-phenethylindolin-2-one* (**33**). Yield: 90%. ^1^H-NMR δ 1.18 (d, 6H, *J* = 7.0 Hz, CH(CH_3_)_2_), 2.85 (ep, 1H, *J* = 7.0 Hz, CH(CH_3_)_2_), 2.92 (t, 2H, *J* = 7.3 Hz, NCH_2_CH_2_), 3.77 (s, 3H, 5-OCH_3_), 3.96 (t, 2H, *J* = 7.3 Hz, NCH_2_CH_2_), 6.83 (dd, 1H, *J* = 8.4 Hz, *J* = 2.6 Hz, H-6), 7.06 (d, 1H, *J* = 8.4 Hz, H-7), 7.14 (d, 1H, *J* = 2.6 Hz, H-4), 7.16–7.28 (m, 7H, H-3′, H-5′, H-2″, H-3″, H-4″, H-5″ and H-6″), 7.36 (d, 2H, *J* = 8.4 Hz, H-2′and H-6′), 12.68 (s, 1H, NNH). ESI-MS *m*/*z* = 412.0 (100%) [M − H]^−^, 436.1 (88%) [M + Na]^+^. IR (KBr): 3431, 2950, 1674, 1557, 1487, 1249, 1160, 1149 cm^−1^. Mp 96–98 °C from ethanol.

*(Z)-3-(2-(4-Isopropylphenyl)hydrazono)-5-methoxy-1-(3-phenylpropyl)indolin-2-one* (**34**). Yield: 70%. ^1^H-NMR δ 1.18 (d, 6H, *J* = 7.0 Hz, CH(CH_3_)_2_), 1.92 (qn, 2H, *J* = 7.3 Hz, NCH_2_CH_2_CH_2_), 2.62 (t, 2H, *J* = 7.3 Hz, NCH_2_CH_2_CH_2_), 2.85 (ep, 1H, *J* = 7.0 Hz, CH(CH_3_)_2_), 3.74–3.79 (m, 2H, NCH_2_CH_2_CH_2_), 3.77 (s, 3H, 5-OCH_3_), 6.85 (dd, 1H, *J* = 8.4 Hz, *J* = 2.4 Hz, H-6), 7.02 (d, 1H, *J* = 8.4 Hz, H-7), 7.14–7.28 (m, 8H, H-4, H-3′, H-5′, H-2″, H-3″, H-4″, H-5″ and H-6″), 7.36 (d, 2H, *J* = 8.4 Hz, H-2′and H-6′), 12.73 (s, 1H, NNH). ESI-MS *m*/*z* = 426.0 (100%) [M − H]^−^, 450.1 (100%) [M + Na]^+^. HRMS (ESI) *m*/*z* [M + H]^+^ calcd for C_27_H_30_N_3_O_2_^+^ 428.2333; found, 428.2332. IR (KBr): 3435, 2957, 1669, 1559, 1485, 1248, 1158 cm^−1^. Hygroscopic compound from dichloromethane/hexane 65:35 (*v*/*v*).

#### 3.1.10. Synthesis of 4-isopropylphenylhydrazonoindolin-2-ones **36**–**39**

4-isopropylphenylhydrazine hydrochloride (1 mmol) was added to a methanolic solution (5 mL) of suitable isatins **35a**–**d** (1 mmol) (prepared using the Sandmayer method [30]). After stirring overnight, the precipitate was filtered and crystallized from ethanol or the reaction mixture was evaporated and the residue purified by column chromatography.

*5-Butyl-3-(2-(4-isopropylphenyl)hydrazono)indolin-2-one* (**36**). Yield: 80%. ^1^H-NMR δ 0.88 (t, 3H, *J* = 7.1, CH_3_CH_2_CH_2_CH_2_) 1.18 (d, 6H, *J* = 7.0 Hz, CH(CH_3_)_2_), 1.29 (sx, 2H, *J* = 7.1 Hz, CH_3_CH_2_CH_2_CH_2_), 1.54 (qn, 2H, *J* = 7.5 Hz, CH_3_CH_2_CH_2_CH_2_), 2.56 (t, 2H, *J* = 7.5 Hz, CH_3_CH_2_CH_2_CH_2_) 2.85 (ep, 1H, *J* = 7.1 Hz, CH(CH_3_)_2_), 6.80 (d, 1H, *J* = 7.9 Hz, H-7), 7.03 (d, 1H, *J* = 7.9, H-6), 7.21 (d, 2H, *J* = 8.7 Hz, H-3′ and H-5′), 7.31–7.34 (m, 3H, H-2′, H-6′ and H-4), 10.88 (s, 1H, NH), 12.72 (s, 1H, NNH). ESI-MS *m*/*z* = 334.0 (100%) [M − H]^−^. IR (KBr): 3298, 2952, 2915, 1683, 1649, 1557, 1513, 1240, 1159 cm^−1^. Mp 164–166 °C from ethanol.

*7-Bromo-3-(2-(4-isopropylphenyl)hydrazono)indolin-2-one* (**37**). Yield: 80%. ^1^H-NMR δ 1.18 (d, 6H, *J* = 7.0 Hz, CH(CH_3_)_2_), 2.86 (ep, 1H, *J* = 7.0 Hz, CH(CH_3_)_2_), 6.98 (t, 1H, *J* = 7.7 Hz, H-5), 7.24 (d, 2H, *J* = 8.4 Hz, H-3′ and H-5′), 7.36–7.41 (m, 3H, H-2′, H-6′ and H-6), 7.52 (d, 1H, *J* = 7.3 Hz, H-4), 11.26 (s, 1H, NH), 12.78 (s, 1H, NNH). ESI-MS *m*/*z* = 355.9 (95%) [M − H]^−^, 357.7 (100%) [M − H]^−^ + 2. HRMS (ESI) *m*/*z* [M + Na]^+^ calcd for C_17_H_16_BrN_3_NaO^+^ 380.0369; found, 380.0378. IR (KBr): 3154, 2959, 1672, 1551, 1170 cm^−1^. Mp 240–242 °C from ethanol.

*Sodium 3-(2-(4-isopropylphenyl)hydrazono)-2-oxoindolin-5-sulfonate* (**38**). Yield: 60%. ^1^H-NMR δ 1.18 (d, 6H, *J* = 7.0 Hz, CH(CH_3_)_2_), 2.85 (ep, 1H, *J* = 7.0 Hz, CH(CH_3_)_2_), 6.83 (d, 1H, *J* = 8.1 Hz, H-7), 7.22 (d, 2H, *J* = 8.6, H-3′ and H-5′), 7.35 (d, 2H, *J* = 8.6 Hz, H-2′ and H-6′), 7.48 (dd, 1H, *J* = 8.1 Hz, *J* = 1.8 Hz, H-6), 7.73 (d, 1H, *J* = 1.1, H-4), 11.04 (s, 1H, NH), 12.69 (s, 1H, NNH). ESI-MS *m*/*z* = 357.8 (100%) [M − Na]^−^. IR (KBr): 3201, 2959, 1560, 1189 cm^−1^. Mp > 250 °C from ethanol.

*(Z)-7-(2-(4-isopropylphenyl)hydrazono)-5H-[1,3]dioxolo[4,5-f]indol-6(7H)-one* (**39**). Yield: 50%. ^1^H-NMR δ 1.17 (d, 6H, *J* = 7.0 Hz, CH(CH_3_)_2_), 2.84 (ep, 1H, *J* = 7.0 Hz, CH(CH_3_)_2_), 5.98 (s, 2H, OCH_2_O), 6.55 (s, 1H, H-4), 7.07 (s, 1H, H-7), 7.20 (d, 2H, *J* = 8.6 Hz, H-3′ and H-5′), 7.29 (d, 2H, *J* = 8.6 Hz, H-2′ and H-6′), 10.79 (s, 1H, NH), 12.59 (s, 1H, NNH). ESI-MS *m*/*z* = 321.9 (100%) [M − H]^−^. IR (KBr): 3177, 1675, 1557, 1464, 1156 cm^−1^. Mp 229–234 °C ethyl acetate/hexane 50:50 (*v*/*v*).

### 3.2. Determination of Llipophilicity by RP-HPLC

The lipophilicity parameters were determined by an RP-HPLC technique [34,35]. Methanol solutions of the investigated hydrazone compounds (0.5 mg/mL) were injected into an Agilent 1260 infinity HPLC system (Agilent Technologies Italia, Milan), equipped with a diode array detector (DAD), and a Phenomenex Kinetex C18 column (150 × 4.6 mm, 5 μ, Phenomenex Italy s.r.l., Castel Maggiore, BO, Italy), and eluted at different mobile phase compositions (0.05 increments of MeOH volume fraction in 10 mM ammonium formate buffer at pH 4.5, ranging ϕ between 0.85 and 0.30). All RP-HPLC measurements were carried out at 25 ± 1 °C, at a flow rate of 1.0 mL/min, and at 254 nm wavelength.

The logarithm of capacity factors (log *k′* = log (t_R_ − t_0_)/t_0_) of each compound at different mobile phase compositions have been calculated; t_R_ represents the retention time of the solute and t_0_ is the column dead time, measured as the elution time of a KNO_3_ solution in MeOH. For each compound, the log *k′* values increased linearly with decreasing MeOH volume fraction. Logarithms of capacity factors extrapolated to the 100% aqueous mobile phase (log *k′_w_*) were calculated from the linear regressions on at least five data points (r^2^ > 0.975).

Lipophilicity was also computationally assessed with the ACDLabs software, release 10.0 (Advanced Chemistry Development, Inc., Toronto, ON, Canada), and BioLoom software, vers. 1.7 (BioByte Corp. Claremont, CA, USA). The SAR equation (1) has been obtained by using the Prism software (ver. 5.01, GraphPad Software Inc., La Jolla, CA, USA).

### 3.3. Inhibition of Aβ_40_ Aggregation

The spectrofluorimetric assays, measuring ThT fluorescence in the presence of Aβ, were done as previously described [22]. Briefly, coincubation samples were prepared as described above (Section 2.2 and Section 2.3) in 96-well black, non-binding microplates (Greiner Bio-One GmbH, Frickenhausen, Germany). Fluorimetric reads were performed in a multiplate reader Infinite M1000 Pro (Tecan, Cernusco sul Naviglio, Italy). Each concentration point was run in triplicate. CD ellipticity measures were performed as detailed in ref. [20], following the increase of the negative band at 215 nm as indicative of β-sheet arrangement.

### 3.4. Cell-Based Assays

Cytoprotection from Aβ_42_-induced neurotoxicity and from ROS generation was assessed in SH-SY5Y cells as already described [22]. At least three independent experiments with six replicates (*n* ≥ 18) were carried out, and the results were averaged. Briefly, for MTT assay, SH-SY5Y cells were cultured in DMEM-Dulbecco’s modified Eagle’s medium (Sigma-Aldrich), grown to 70% confluence and seeded for experiment in 96-well plates at a density of 10,000 cells/well. Cultured cells were added with a DMEM solution of Aβ_42_, alone or in mixture with 28 (5 μM final concentration of both) and incubated at 37 °C in 5% CO_2_. At the time points of 24 and 48 h, the culture medium was replaced by DMEM supplemented with a solution of MTT in PBS (50 μg/mL final concentration). After removal of solution and addition of DMSO, the absorbance of product formazan produced from MTT was measured at 570 nm using a multilabel plate counter Victor3 V (Perkin Elmer, Milan, Italy), with DMSO medium as the blank solution.

Intracellular ROS production was evaluated using an oxidation-sensitive fluorescent probe, 2′,7′-dichlorodihydrofluorescin diacetate (DCFH-DA; Sigma-Aldrich). Viable SHSY5Y cells were seeded in a black 96-well cell culture plate for 24 h, then the cells were incubated in DMEM supplemented with different concentration (0 to 20 μM) of **28** for 1 h. After washing, DCFH-DA (50 μM final concentration) was added directly to each well, and the plate was incubated at 37 °C in 5% CO_2_ for 0.5 h. After washing, 100 μM H_2_O_2_/well in DMEM was added and the cells were incubated for an additional 30 min. The formation of fluorescent dichlorofluorescein (DCF) due to oxidation of DCFH in the presence of ROS was read directly in each well at an excitation wavelength of 485 nm and an emission wavelength of 530 nm using a multilabel plate reader Victor3 V (Perkin Elmer), with DMSO medium as the control.

## 4. Conclusions

The SAR studies on the indoline-2-one 3-arylhydrazone derivatives reported herein warranted further gain of knowledge in our ongoing study of inhibition of Aβ aggregation. Rooting on previous results [19,20,21] and keeping phenylhydrazone **1** [22] as a hit compound prone to optimization, the focused exploration of substituents on the indole scaffold, the modifications on the hydrazone linker, and the isosteric replacement of the phenyl of hydrazone moiety with thiazole, highlighted the essential role of 5-methoxy substituent, the requisite of an optimal lipophilicity, and the opportunity of alkylating the indole nitrogen for maintaining an efficient inhibition of Aβ aggregation. In this regard, a handful of derivatives (thiazolyl hydrazone **15** and *N*^1^-substituted indolin-2-ones **26**, **28**, **32**–**34**) were found almost equipotent in vitro with the hit compound 1. Among them, compound **28**, i.e., the *N*^1^-cyclopropyl derivative of 1, emerged from cell-based assays as a multitarget agent with strong cytoprotective effects in both Aβ_42_ oligomer toxicity and oxidative stress. These results were in full agreement with those already reported for congeners of 28, particularly for cytoprotection from amyloid insult that resulted well comparable with reference compound **1** [22]. Aβ antifibrillogenic data have been analyzed to disclose reliable QSARs, and a bilinear correlation between pIC_50_ and log *k’*_w_ determined by RP-HPLC was derived which hold for those compounds that do not undergo detrimental steric effects in binding Aβ peptides.

The present work adds new information to the potential of indolin-2-one 3-arylhydrazones as privileged structures for the inhibition of Aβ aggregation and neurotoxicity, and pleiotropic agents for AD therapy. Despite the claims of disengagement of pharmaceutical industry from AD research [42], because of the controversial results so far obtained in clinical trials, many programs from research institutions continue to bet in the so-called amyloid hypothesis. In this light, the multitarget activity of **28** could deserve for this compound a preliminary pharmacological investigation in animal models of Aβ-dependent neurodegeneration, provided that further efforts in ameliorating aqueous solubility would be beneficial. The potency and versatility of indolin-2-one derivatives may still encounter the interest of research and development of small molecules as pharmacological tools for treating AD and related neurodegenerative syndromes.

## Figures and Tables

**Table 1:** Aβ Antiaggregating Activity of Compounds **1** and **4**–**11**.

	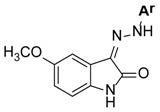	
Comp.	Ar	IC_50_ (µM) or (% Inhibition @ 100 µM) ^1^
**1**	4-(CH(CH_3_)_2_)C_6_H_4_	0.43 ± 0.04
**4**	4-CNC_6_H_4_	(16 ± 3)
**5**	4-CF_3_C_6_H_4_	(57 ± 5)
**6**	3,5-(CF_3_)_2_C_6_H_3_	9.9 ± 0.8
**7**	Pyrid-2-yl	90 ± 5
**8**	1-Naphthyl	28 ± 2
**9**	Quinolin-2-yl	(67 ± 4)
**10**	Quinolin-8-yl	(58 ± 4)
**11**	7-Cl-quinolin-4-yl	(14 ± 2)

^1^ Data are mean ± SEM of three independent experiments.

**Table 2:** Aβ Antiaggregating Activity of Compounds **13**–**18**.

	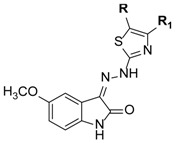		
Comp.	R	R_1_	IC_50_ (µM) or (% Inhibition @ 100 µM) ^1^
**13**	H	CH_2_Cl	13 ± 3
**14**	CH_3_	CH_3_	(43 ± 1)
**15**	H	C_6_H_5_	1.2 ± 0.2
**16**	H	3′-OHC_6_H_4_	16 ± 2
**17**	H	3′-OCH_3_C_6_H_4_	34 ± 6
**18**	H	3′-BrC_6_H_4_	40 ± 2

^1^ Data are mean ± SEM of three independent experiments.

**Table 3:** Aβ Antiaggregating Activity of Compounds **19** and **21**–**23.**

	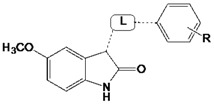		
Comp.	R	L	IC_50_ (µM) or (% inhibition @ 100 µM) ^1^
**19**	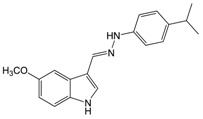		41 ± 3
**21**	4-CH(CH_3_)_2_	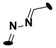	6.7 ± 0.2
**22**	3-Cl	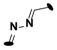	11 ± 2
**23**	3-Cl	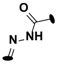	(31 ± 3)

^1^ Data are mean ± SEM of three independent experiments.

**Table 4:** Aβ Antiaggregating Activity of Compounds **24**, **26**, **28** and **32**–**34**.

	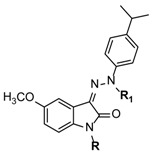		
Comp.	R	R_1_	IC_50_ (µM) ^1^
**24**	CH_3_	CH_3_	11 ± 2
**26**	CH_3_	H	0.81 ± 0.11
**28**	Cyclopropyl	H	0.80 ± 0.13
**32**	Benzyl	H	0.53 ± 0.10
**33**	2-Phenylethyl	H	0.61 ± 0.03
**34**	3-Phenylpropyl	H	0.83 ± 0.20

^1^ Data are mean ± SEM of three independent experiments.

**Table 5:** Aβ Antiaggregating Activity of Compounds **36**–**39**.

	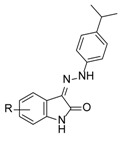	
Comp.	R	% Inhibition @ 100 µM ^1^
**36**	5-*n*-Butyl	55 ± 1
**37**	7-Br	<5
**38**	5-SO_3_^−^Na^+^	35 ± 4
**39**	5,6-methylenedioxy	70 ± 4

^1^ Data are mean ± SEM of three independent experiments.

**Table 6:** Aβ antiaggregating potencies and physicochemical descriptors of isatin hydrazones.

Comp.	pIC_50_	log *k’*_w_	CMR	MV
**1**	6.40	5.44	8.288	2.304
**6**	5.00	6.29	8.475	2.657
**7**	4.05	2.34	7.369	1.942
**8**	4.55	5.15	9.113	2.413
**13**	4.89	3.95	8.149	2.019
**15**	5.92	5.10	9.738	2.445
**16**	4.80	4.58	9.824	2.417
**17**	4.47	5.29	10.320	2.661
**18**	4.40	5.67	10.494	2.570
**19**	4.39	6.42	9.331	2.681
**21**	5.17	4.82	9.331	2.681
**22**	4.96	4.21	8.445	2.309
**24**	4.96	4.23	9.935	2.946
**26**	6.09	5.96	9.402	2.733
**28**	6.10	6.36	10.088	2.778
**32**	6.28	7.44	11.931	3.421
**33**	6.21	7.24	12.392	3.581
**34**	6.08	7.87	12.853	3.741

## Supplementary Materials

The following are available online at http://www.mdpi.com/1420-3049/23/7/1544/s1, Table S1: Experimental lipophilicity as described by RP-HPLC log *k*’_w_, and predicted 1-octanol–water partition coefficients of the new synthesized isatin hydrazone derivatives; Figure S1: Log *k’*_w_ versus log P calculated values by means of ACDLabs suite; Figure S2: Scatter plot of Aβ aggregation inhibition potency (pIC_50_) versus calculated molar refractivity.

## References

[1] Alzheimer’s Association (2016). 2016 Alzheimer’s Disease Facts and Figures. Alzheimer’s Dement..

[2] Anand P., Singh B. (2013). A Review on Cholinesterase Inhibitors for Alzheimer’s Disease. Arch. Pharm. Res..

[3] Lo D., Grossberg G.T. (2011). Use of Memantine for the Treatment of Dementia. Expert Rev. Neurother..

[4] Chiti F., Dobson C.M. (2017). Protein Misfolding, Amyloid Formation, and Human Disease: A Summary of Progress over the Last Decade. Annu. Rev. Biochem..

[5] Benilova I., Karran E., De Strooper B. (2012). The toxic Aβ oligomer and Alzheimer’s disease: An emperor in need of clothes. Nat. Neurosci..

[6] Lambert M.P., Barlow A.K., Chromy B.A., Edwards C., Freed R., Liosatos M., Morgan T.E., Rozovsky I., Trommer B., Viola K.L. (1998). Diffusible, nonfibrillar ligands derived from Abeta1-42 are potent central nervous system neurotoxins. Proc. Natl. Acad. Sci. USA.

[7] Walsh D.M., Klyubin I., Fadeeva J.V., Cullen W.K., Anwyl R., Wolfe M.S., Rowan M.J., Selkoe D.J. (2002). Naturally secreted oligomers of amyloid beta protein potently inhibit hippocampal long-term potentiation in vivo. Nature.

[8] Shankar G.M., Li S., Mehta T.H., Garcia-Munoz A., Shepardson N.E., Smith I., Brett F.M., Farrell M.A., Rowan M.J., Lemere C.A. (2008). Amyloid-beta protein dimers isolated directly from Alzheimer’s brains impair synaptic plasticity and memory. Nat. Med..

[9] Cummings J., Lee G., Mortsdorf T., Ritter A., Zhong K. (2017). Alzheimer’s disease drug development pipeline: 2017. Alzheimer’s Dement. (N. Y.).

[10] Levine H. (2007). Small molecule inhibitors of Abeta assembly. Amyloid.

[11] Ramos E., Egea J., de Los Ríos C., Marco-Contelles J., Romero A. (2017). Melatonin as a versatile molecule to design novel multitarget hybrids against neurodegeneration. Future Med. Chem..

[12] Török M., Abid M., Mhadgut S.C., Török B. (2006). Organofluorine inhibitors of amyloid fibrillogenesis. Biochemistry.

[13] Cohen T., Frydman-Marom A., Rechter M., Gazit E. (2006). Inhibition of amyloid fibril formation and cytotoxicity by hydroxyindole derivatives. Biochemistry.

[14] Gsponer J., Haberthur U., Caflisch A. (2003). The role of side-chain interactions in the early steps of aggregation: Molecular dynamics simulations of an amyloid-forming peptide from the yeast prion Sup35. Proc. Natl. Acad. Sci. USA.

[15] Hills R.D., Brooks C.L. (2007). Hydrophobic cooperativity as a mechanism for amyloid nucleation. J. Mol. Biol..

[16] Convertino M., Pellarin R., Catto M., Carotti A., Caflisch A. (2009). 9,10-Anthraquinone hinders beta-aggregation: How does a small molecule interfere with Abeta-peptide amyloid fibrillation?. Protein Sci..

[17] Zheng J., Ma B., Nussinov R. (2006). Consensus features in amyloid fibrils: Sheet-sheet recognition via a (polar or nonpolar) zipper structure. Phys. Biol..

[18] Cellamare S., Stefanachi A., Stolfa D.A., Basile T., Catto M., Campagna F., Sotelo E., Acquafredda P., Carotti A. (2008). Design, Synthesis, and Biological Evaluation of Glycine-based Molecular Tongs as Inhibitors of Abeta1-40 Aggregation in Vitro. Bioorg. Med. Chem..

[19] Catto M., Aliano R., Carotti A., Cellamare S., Palluotto F., Purgatorio R., De Stradis A., Campagna F. (2010). Design, synthesis and biological evaluation of indane-2-arylhydrazinylmethylene-1,3-diones and indol-2-aryldiazenylmethylene-3-ones as beta-amyloid aggregation inhibitors. Eur. J. Med. Chem..

[20] Campagna F., Catto M., Purgatorio R., Altomare C.D., Carotti A., De Stradis A., Palazzo G. (2011). Synthesis and biophysical evaluation of arylhydrazono-1*H*-2-indolinones as β-amyloid aggregation inhibitors. Eur. J. Med. Chem..

[21] Catto M., Arnesano F., Palazzo G., De Stradis A., Calò V., Losacco M., Purgatorio R., Campagna F. (2014). Investigation on the Influence of (Z)-3-(2-(3-Chlorophenyl)hydrazono)-5,6-dihydroxyindolin-2-one (PT2) on β-amyloid(1-40) Aggregation and Toxicity. Arch. Biochem. Biophys..

[22] Pisani L., De Palma A., Giangregorio N., Miniero D.V., Pesce P., Nicolotti O., Campagna F., Altomare C.D., Catto M. (2017). Mannich base approach to 5-methoxyisatin 3-(4-isopropylphenyl)hydrazone: A water-soluble prodrug for a multitarget inhibition of cholinesterases, beta-amyloid fibrillization and oligomer-induced cytotoxicity. Eur. J. Pharm. Sci..

[23] Manoharan S., Guillemin G.J., Abiramasundari R.S., Essa M.M., Akbar M., Akbar M.D. (2016). The Role of Reactive Oxygen Species in the Pathogenesis of Alzheimer’s Disease, Parkinson’s Disease, and Huntington’s Disease: A Mini Review. Oxid. Med. Cell. Longev..

[24] Crestini C., Saladino R. (1994). A New Efficient and Mild Synthesis of 2-Oxindoles by One-Pot Wolff-Kishner Like Reduction of Isatin Derivatives. Synth. Commun..

[25] Kondratieva M.L., Pepeleva A.V., Belskaia N.P., Koksharov A.V., Groundwater P.V., Robeyns K., Van Meervelt L., Dehaen W., Fan Z.J., Bakulev V.A. (2007). A New Synthetic Method for the 2*H*-[1,2,3] Thiadiazolo[5,4-b]indoles. Tetrahedron.

[26] Kumar V., Sharma S.K., Singh S., Kumar A., Sharma S. (2010). Synthesis and evaluation of novel indolylthiadiazinoazetidinones and indolylthiadiazinothiazolidinones as antimicrobial agents. Arch. Pharm..

[27] Muzalevskiy V.M., Balenkova E.S., Shastin A.V., Magerramov A.M., Shikhaliev N.G., Nenajdenko V.G. (2011). New method for the preparation of 3-diazo-1,3-dihydroindol-2-ones. Russ. Chem. Bull..

[28] Yoshino T., Shibata K., Wada H., Bando Y., Ishikawa K., Takezoe H., Mori T. (2009). Organic field-effect transistors based on solution-processible dibenzotetrathiafulvalene derivatives. Chem. Lett..

[29] Lackey K., Sternbach D.D. (1993). Synthesis of substituted quinoline-4-carboxylic acids. Synthesis.

[30] Sandmeyer T. (1919). Über Isonitrosoacetanilide und deren Kondensation zu Isatinen. Helv. Chim. Acta.

[31] Dahiya R., Narayan S., Bindal V., Kumar V., Handa R.N., Pujari H.K. (1987). Heterocyclic Systems Containing Bridgehead Nitrogen Atom: Part LX—Synthesis of Thiazolo<2′,3′:3,4><1,2,4>triazino<5,6-b>indoles. Indian J. Chem. Sect. B.

[32] Gui Q., Dai F., Liu J., Chen P., Yang Z., Chen X., Tan Z. (2014). Synthesis of N-alkyl isatins via oxidative cyclization of *N*-alkyl 2-bromo(chloro)acetanilides. Org. Biomol. Chem..

[33] Vyas D.J., Fröhlich R., Oestreich M. (2010). Stereochemical surprises in the Lewis acid-mediated allylation of isatins. J. Org. Chem..

[34] Soto-Otero R., Mendez-Alvarez E., Sanchez-Iglesias S., Zubkov F.I., Voskressensky L.G., Varlamov A.V., de Candia M., Altomare C.D. (2008). Inhibition of 6-hydroxydopamine-induced oxidative damage by 4,5-dihydro-3*H*-2-benzazepine *N*-oxides. Biochem. Pharmacol..

[35] Soto-Otero R., Mendez-Alvarez E., Sanchez-Iglesias S., Labandeira-García J.L., Rodríguez-Pallares J., Zubkov F.I., Zaytsev V.P., Voskressensky L.G., Varlamov A.V., de Candia M. (2012). 2-Benzazepine nitrones protect dopaminergic neurons against 6-hydroxydopamine-induced oxidative toxicity. Arch. Pharm..

[36] Tan L.C., Carr P.-W., Fréchet J.M., Smigol V. (1994). Liquid chromatographic study of solute hydrogen bond basicity. Anal. Chem..

[37] Parisi G., Degennaro L., Carlucci C., de Candia M., Mastrorilli P., Roller A., Holzer W., Altomare C.D., Pace V., Luisi R. (2017). A greener and efficient access to substituted four- and six-membered sulfur-bearing heterocycles. Org. Biomol. Chem..

[38] Tan L.C., Carr P.-W. (1993). Extra-thermodynamic relationships in chromatography-study of the relationship between the slopes and intercepts of plots of log *k*’ vs. mobile phase composition in reversed-phase chromatography. J. Chromat. A.

[39] Kubinyi H. (1977). Quantitative structure-activity relationships. 7. The bilinear model, a new model for nonlinear dependence of biological activity on hydrophobic character. J. Med. Chem..

[40] Berridge M.V., Tan A.S. (1993). Characterization of the cellular reduction of 3-(4,5-dimethylthiazol-2-yl)-2,5-diphenyltetrazolium bromide (MTT): Subcellular localization, substrate dependence, and involvement of mitochondrial electron transport in MTT reduction. Arch. Biochem. Biophys..

[41] Wang H., Joseph J.A. (1999). Quantifying cellular oxidative stress by dichlorofluorescein assay using microplate reader. Free Radical Biol. Med..

[42] Hawkes N. (2018). Pfizer abandons research into Alzheimer’s and Parkinson’s diseases. BMJ.

