# Supplementary material for: Insights into Structure-Activity Relationships of 3-Arylhydrazonoindolin-2-One Derivatives for Their Multitarget Activity on β-Amyloid Aggregation and Neurotoxicity
Source: Molecules. 2018 Jun 26;23(7):1544. doi: 10.3390/molecules23071544 (PMC6100259; doi:10.3390/molecules23071544)
Supplement: Supplementary file 1 [file molecules-23-01544-s001.pdf]

Insights into structure-activity relationships of 3-arylhydrazonoindolin-2-one derivatives for their multitarget activity on beta amyloid aggregation and neurotoxicity

Rosa Purgatorio,[1] Modesto de Candia,[1] Annalisa De Palma,[2] Francesco De Santis,[2] Leonardo Pisani,[1] Francesco Campagna,[1] Saverio Cellamare,[1] Cosimo D. Altomare,[1] Marco Catto[1,*]

[1] Dipartimento di Farmacia-Scienze del Farmaco, Università degli Studi di Bari "Aldo Moro", via E. Orabona 4, I-70125 Bari (Italy); rosa.purgatorio@uniba.it; modesto.decandia@uniba.it; leonardo.pisani@uniba.it; francesco.campagna51@gmail.com; saverio.cellamare@uniba.it; cosimodamiano.altomare@uniba.it

[2] Dipartimento di Bioscienze, Biotecnologie e Biofarmaceutica, Università degli Studi di Bari "Aldo Moro", via E. Orabona 4, I-70125 Bari (Italy); annalisa.depalma@uniba.it; desantis.nutrizione@gmail.com

* Correspondence: marco.catto@uniba.it; Tel.: +39-080-544-2780

SUPPLEMENTARY MATERIALS

Lipophilicity measurements and calculations

Lipophilicity parameters of 26 isatin-containing hydrazone derivatives were experimentally determined through RP-HPLC. Methanol solutions of the compounds (0.5 mg/mL) were injected into HPLC equipped with a Kinetex C18 column (150 × 4.6 mm, 5μ) from Phenomenex (Phenomenex Italy s.r.l., Castel Maggiore, BO, Italy), and eluted at different mobile phase composition (0.05 increments of MeOH volume fraction in 10 mM ammonium formate buffer at pH 4.5, ranging between 0.85 and 0.30). All the RP-HPLC measurements were carried out at 25 ± 1 °C, flow rate of 1.0 mL/min, and 254 nm wavelength, on an Agilent 1260 infinity HPLC system (Agilent Technologies Italia, Milan), equipped with a diode array detector (DAD).

The logarithm of capacity factors (log *k*′) of each compound at different mobile phase compositions have been calculated as:

$\log k' = \log (t_R - t_0)/t_0$

where t_R is the retention time of the solute and t_0 is the column dead time, measured as the elution time of a solution of KNO_3 in MeOH.

For each compound, log *k*′ values increased linearly with decreasing MeOH volume fraction (φ), and the logarithm of the capacity factor extrapolated to 100% aqueous mobile phase (log k'_w) was calculated from the following linear correlation obtained from regression analysis of at least five data points ($r^2 > 0.975$):

$\log k' = \log k'_w - S \cdot \varphi$

In the above equation, log k'_w and *S* represent the intercept and slope values, respectively, of the linear correlations. The RP-HPLC data are reported for each investigated compound in Table S1.

Partition coefficients in *n*-octanol/water system (log P) were calculated with ACDLabs software, release 10.0 (Advanced Chemistry Development, Inc., Toronto, Canada); Log P values are also reported in Table S1.

Table S1. Experimental lipophilicity as described by RP-HPLC log k'_w, and predicted 1-octanol–water partition coefficients of the new synthesized isatin-containing hydrazone derivatives.

Comp	Log k'_w[a]	slope[a]	Log P_{ACD}[b]
**1**	5.442	5.9010	4.20
**4**	4.028	4.9688	3.05
**5**	5.425	6.1207	4.13
**6**	6.287	6.7665	6.02
**7**	2.344	3.4123	1.77
**8**	5.149	5.5058	4.35
**9**	3.715	5.2443	3.12
**10**	4.668	5.2624	4.12
**11**	4.358	4.6670	4.24
**13**	3.953	4.8485	2.88
**14**	4.262	4.9042	3.53
**15**	5.103	5.7110	4.37
**16**	4.584	5.5138	3.62
**17**	5.292	5.9244	4.13
**18**	5.670	6.0519	5.04
**19**	6.422	5.3720	3.89
**21**	4.823	5.5870	4.97
**22**	4.215	5.0771	4.43
**23**	3.902	4.7747	3.53
**24**	4.231	5.0336	4.18
**26**	5.957	6.2528	4.22
**28**	6.308	6.5643	4.58
**32**	7.435	7.6758	5.99
**33**	7.238	7.8335	6.41
**34**	8.296	8.4185	6.84
**39**	5.617	6.0443	3.70

[a]Intercept (log k'_w) and slope (S) of the linear correlation between log k' (capacity factor) and MeOH volume fraction (φ) in the aqueous mobile phases, determined for each compound by RP-HPLC. [b]*n*-Octanol/water partition coefficient, calculated by ACD Labs software, release 10.0 (Advanced Chemistry Development, Inc., Toronto, Canada), and CLOGP software version 4.73 (Biobyte, Claremont, CA, USA).

The lipophilicity experimental values were correlated (Fig. S1) with those calculated by ACDLabs software suite. The linear equation correlating log k'_w and CLOGP has a slope close to one (+ 1.03) and an intercept of about + 0.70, the latter revealing a systematic positive deviation from linearity, likely due to silanophilic interactions on the silica-based C18 stationary phase.

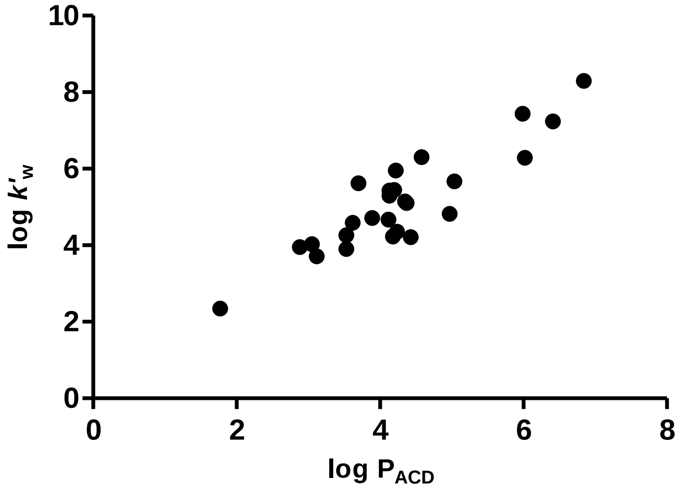

Figure S1. Log k'_w versus log P calculated values by means of ACDLabs suite:

$$\log k'_w = 1.031 \log P_{ACD} + 0.701 \ (n = 25, r^2 = 0.805).$$

Based on the values reported in Table 6 of the paper, a bilinear correlation was also found between pIC_{50} and molar refractivity descriptor calculated by the ACDLabs software (Fig. S2). In the better equation compounds **3**, **8**, **16-19** and **24** were omitted.

$$pIC_{50} = 1.136 \ (\pm 0.65) \ CMR - 0.797 \ (\pm 0.65) \log (\beta CMR + 1) + 0.86$$

$n = 11$, $r^2 = 0.704$, $s = 0.479$, $\log \beta = -1.657$

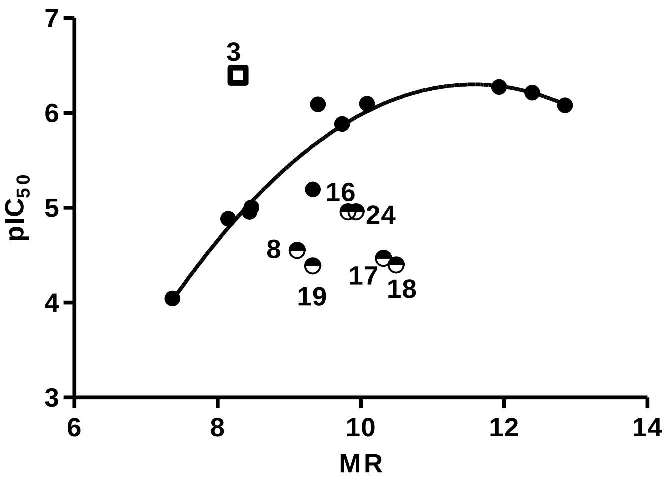

Figure S2. Scatter plot of Aβ aggregation inhibition potency (pIC_{50}) versus calculated molar refractivity

(CMR, ACDLabs suite, vers. 10).
